# The constraint of ignoring the subtidal water climatology in evaluating the changes of coralligenous reefs due to heating events

**DOI:** 10.1038/s41598-020-74249-9

**Published:** 2020-10-15

**Authors:** Giulia Ceccherelli, Federico Pinna, Arianna Pansini, Luigi Piazzi, Gabriella La Manna

**Affiliations:** 1 Department of Chemistry and Pharmacy, University of Sassari, via Piandanna 4, 07100 Sassari, Italy; 2 MareTerra Onlus—Environmental Research and Conservation, 07041 Alghero, SS Italy

**Keywords:** Climate-change ecology, Community ecology, Conservation biology

## Abstract

Predicting community-level responses to seawater warming is a pressing goal of global change ecologists. How far such predictions can be derived from a fine gradient of thermal environments needs to be explored, even if ignoring water climatology does not allow estimating subtidal marine heat waves. In this study insights about the influence of the thermal environment on the coralligenous community structure were gained by considering sites (Sardinia, Italy) at different temperature conditions. Heating events were measured (by loggers at 18 m, 23 m, 28 m, 33 m and 38 m deep) and proxies for their duration (the maximum duration of events warmer than the 90th percentile temperature), intensity (the median temperature) and variability (the number of daily ΔT larger than the mean daily ΔT, and the number of heating events larger in ΔT than the 90th percentile ΔT) were selected by GAM models. Reliable predictions of decrease in coralligenous richness of taxa/morphological groups, with relevant increment in turfs and encrusting coralline algae abundance at the expenses of bryozoans were made. Associations to the different types of heating descriptor have highlighted the aspect (intensity, duration or variability) of the heating events and the threshold for each of them responsible for the trajectories of change.

## Introduction

Predicting community-level responses to sea water warming is a pressing goal of global change ecologists. A large amount of studies have reported that this pressure is exerting an overwhelming impact on marine ecosystems worldwide^1–4^, revealing that the majority of the changes are occurring rapidly with an increased risk of sudden nonlinear transformations^5^. Understanding the response to warming of marine communities is of critical importance because of their complexity, as often they comprise highly divergent taxa (from microbes to metazoans), different phyla, trophic levels and functional groups^6,7^, and because warming may affect differently the processes leading to unpredictable shifts in the structure and function of the ecosystem.

The speed of climate change has become a more important driver of change in biodiversity this century, leading to an accelerating rate of species loss^8–11^. Indeed, the thermal stress to which marine communities are subjected may not be able to keep up in terms of adaptation with important consequences on biodiversity and the economy associated. Warming, through both gradual increases in temperature and short-term extreme events, influences processes across all biological scales and can lead to step-wise shifts in ecosystem structure and functioning^12–14^. Concurrent with long-term persistent warming, discrete prolonged periods of anomalously warm seawater (i.e. marine heat waves MHWs, periods of at least five consecutive days of water temperatures exceeding the 90th percentile according to the local climatology^15^) have increased significantly in frequency and duration since the early twentieth century^16^, with the projection to further increase for the anthropogenic climate change^17^. Several regions on the Globe seem particularly vulnerable to MHWs intensification due to the coexistence of high levels of biodiversity, a prevalence of species found at their warm range edges or concurrent non-climatic human impacts^18^: in these areas MHWs have deleterious impacts across a range of biological processes and taxa, including critical foundation species such as corals^19,20^, seagrasses^21–23^ and kelps^24,25^.

In the Mediterranean Sea, the coralligenous reef is one of the most important benthic ecosystems, whose conservation is a major challenge for the management of coastal marine systems^26^. Coralligenous reefs are hot-spots of biodiversity^27^ being produced by carbonate deposition of encrusting coralline algae (*Lithophyllum*, *Lithothammnium*, and *Mesophyllum*) and by several invertebrates, mostly Cnidaria, Polychaeta, Porifera and Bryozoa^28,29^. Coralligenous reefs are habitats of European Community interest^30^ due to the ecological services they provide for fisheries and their important role in the balance of CO_2_^26–28,31,32^ and to their vulnerability to anthropogenic local stressors, such re-suspension of nearby sediments, eutrophication due to wastewater discharge or aquaculture, direct habitat destruction through fishing, anchoring, and coastal construction activities (including submarine cables)^28,33–35^. At a wider scale, climate change is also known to affect the coralligenous reef by increasing the incidence of warming events with direct and indirect consequences on several species^36–38^. In particular, mortalities of gorgonians have been recorded in recent decades^36,39,40^ and have been linked to different water heating events, during water stratification seasons^37,40–42^. Overall, thermal tolerance has been highlighted mainly for gorgonians, bryozoans and sponges species, and lethal and sublethal responses (e.g. necrosis or decreased polyps activity) to the exceeding of heating thresholds or to manipulated duration of heating events have been identified^43–45^. Overall, the survival horizon of specific taxa as well as of the whole coralligenous reef is considered strongly dependent on heat waves occurrence^46^.

In general, the assessment of vulnerability of marine habitats to warming has been mainly based on changes in satellite-derived sea-surface temperature (SST) and recently on detection of MHWs, identified as SST anomalies and categorized for intensity, duration and frequency based on the local climatology^12,47–49^. Satellite-derived SST is undoubtedly a useful tool for subtidal habitats and its usefulness as proxy for very shallow water temperature is allowed; however, depending on the ecological questions to ask, benthic ecologists are encouraged to use loggers to rely on accurate and precise temperature estimates, as deep water temperature can be scarcely predicted from SST^50,51^. The use of loggers pose a great limit on the testable hypotheses in marine ecology, as if on the one hand we can deal with reliable measurements, on the other hand we cannot know the water climatology at the desired depth. As a matter of fact most of the investigations testing the effect of warming on subtidal species have manipulated the temperature in mesocosms either testing a wide range of tolerable condition^52,53^ or reproducing field conditions measured from loggers^54^, but ignoring the climatology at the depth of interest and thus the temperature anomalies. This scenario prevents the possibility of detecting and categorizing the real subtidal MHWs for any subtidal locality.

**Figure 1:**
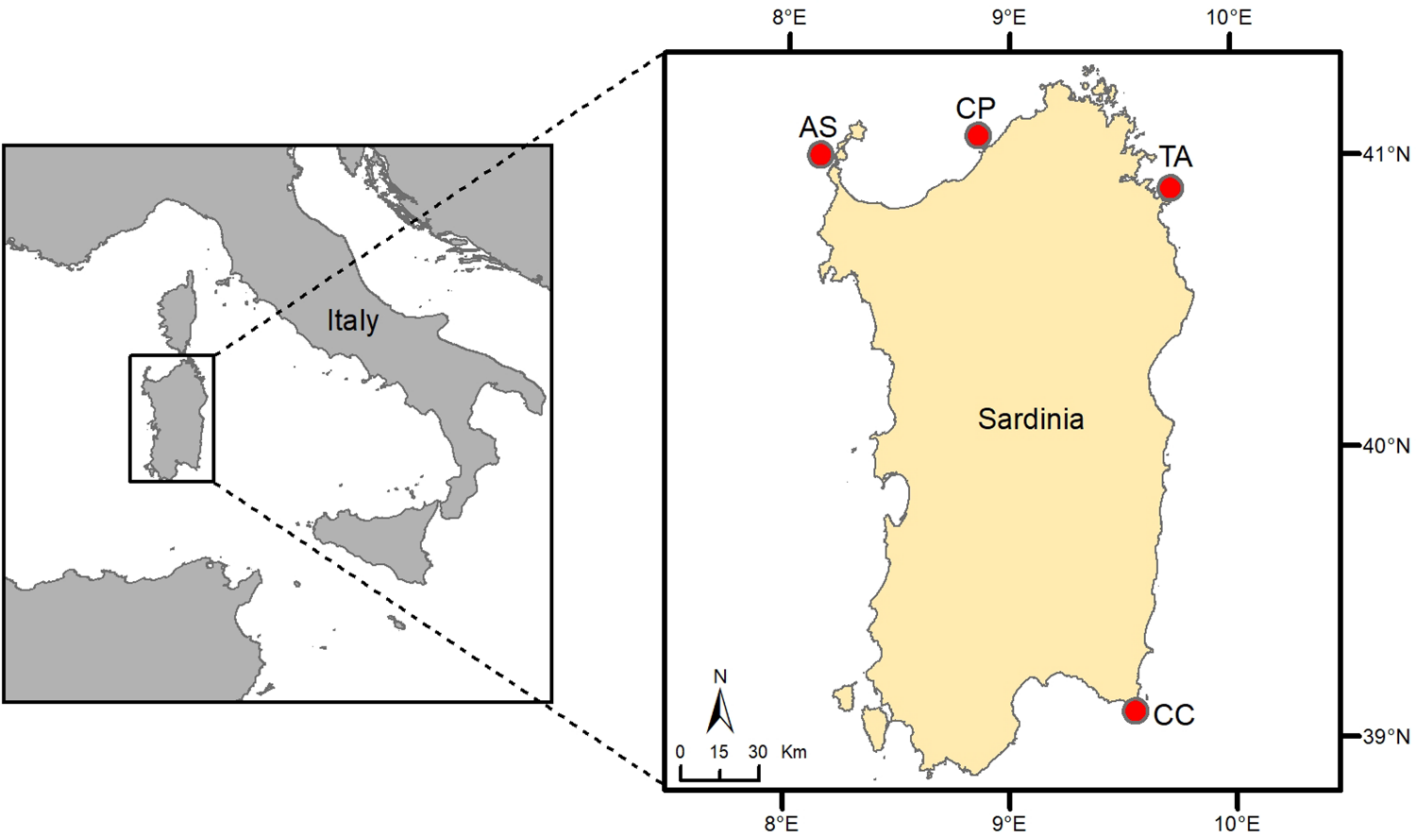
The study sites (AS, CP, TA and CC) in Sardinia (western Mediterranean): AS ‘Isola dell’Asinara MPA’, CP ‘Costa Paradiso’, TA ‘Tavolara Punta Coda Cavallo MPA’ and CC ‘Capo Carbonara MPA’.

**Figure 2:**
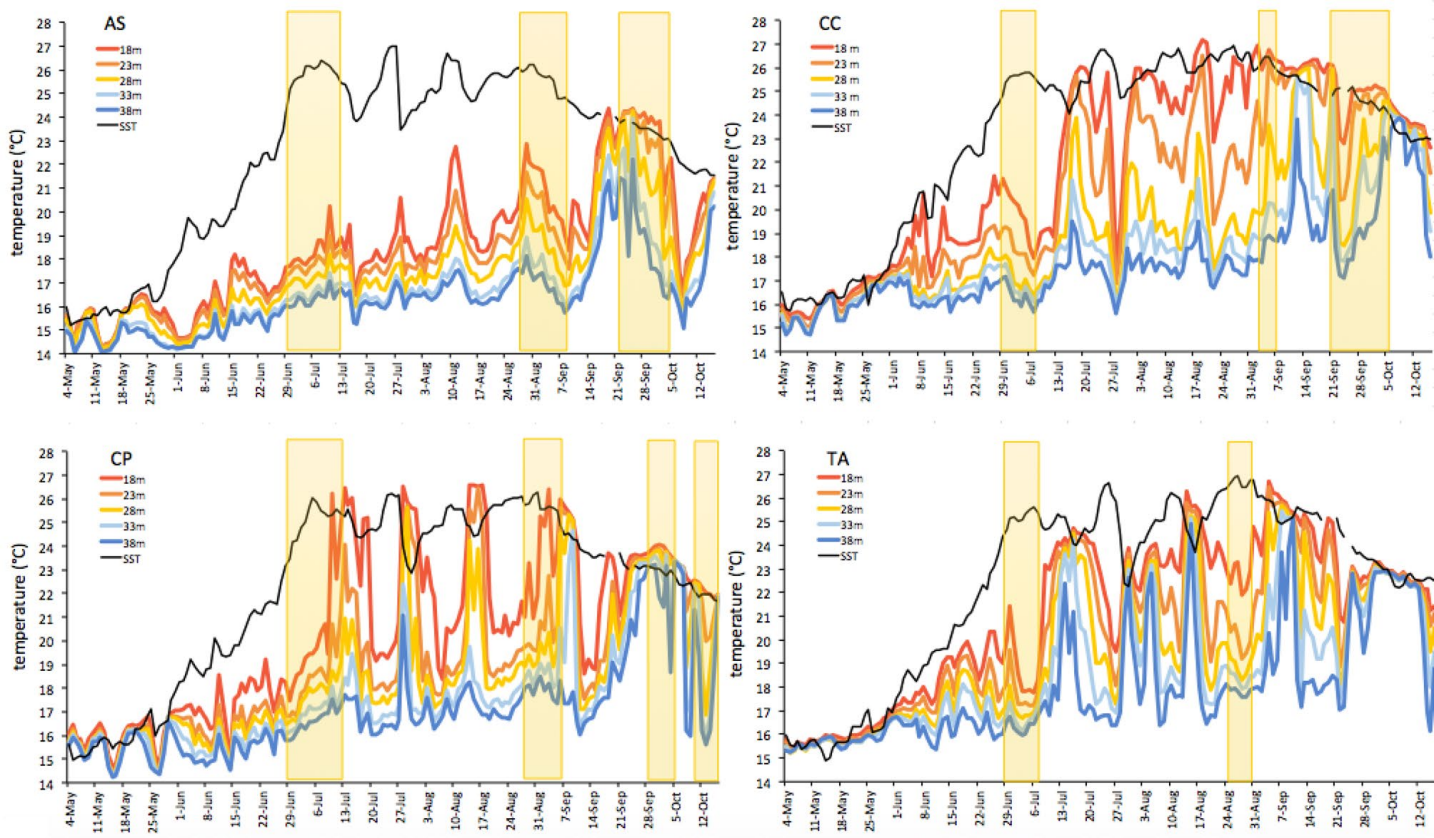
Daily temperature from May 4 to October 16 2019 at the four sites (AS, CP, TV, and CC), at each depth (from loggers, at 18 m, 23 m, 28 m, 33 m and 38 m). In black SST (from satellites). Yellow squared areas indicate MHWs (categorized in Table S1, supplementary 1).

The present study was carried out from May to October 2019 in four sites of Sardinia Island (Western Mediterranean, Fig. 1): Isola dell’Asinara Marine Protected Area (AS), Costa Paradiso (CP), Tavolara Punta Coda Cavallo Marine Protected Area (TA) and Capo Carbonara Marine Protected Area (CC). All the sites are far from any anthropogenic source of influence (i.e. urbanization, industries, aquaculture, harbors, mining and dredging) and are characterized by high level of oligotrophy. All of them support high diverse coralligenous reefs, which are coveted destinations of scuba tourists^55^. Recent investigations have provided evidence that the assemblage structure (taxa composition and relative abundance) of these coralligenous reefs is strongly dependent on the combination of site and depth^56^, which might correspond to the specific thermal environment (Fig. 2). In the present work, temperature conditions (during water stratification May–October 2019) associated with site × depth context were measured by loggers so to identify several descriptors of the duration, intensity and variability of heating events and to test their influence on the coralligenous community structure. The aim was twofold: the first was to evaluate if such heating descriptors can be useful predictors of the coralligenous reef community structure, while the second was to identify the structure and the taxa of the community associated to the current heating events, so to provide tools to draw the trajectories of change of this community due to future warming scenarios. The results may contribute to identifying heating descriptors relevant to any kind of subtidal habitat vulnerability, for which MHWs metrics cannot be applied for the lack of climatological multi-year data.

## Results

### Thermal regimes at the sites and heating descriptors

Important differences in the thermal regimes among sites depending on the depth were evidenced by graphical inspection (Fig. 2). AS was the site with the least differences in temperature among depths and the coolest maximum temperature at the shallower depth (24.36 °C at 18 m), while CP was the site with the largest variations in temperature (at the least down to 28 m). CC was the site with the largest differences in temperature among depths and TA was the site where water stratification was complete, as at the end of the summer temperature was homogeneous from the surface down 38 m deep (Fig. 2 and Fig S1, supplementary 1).

**Figure 3:**
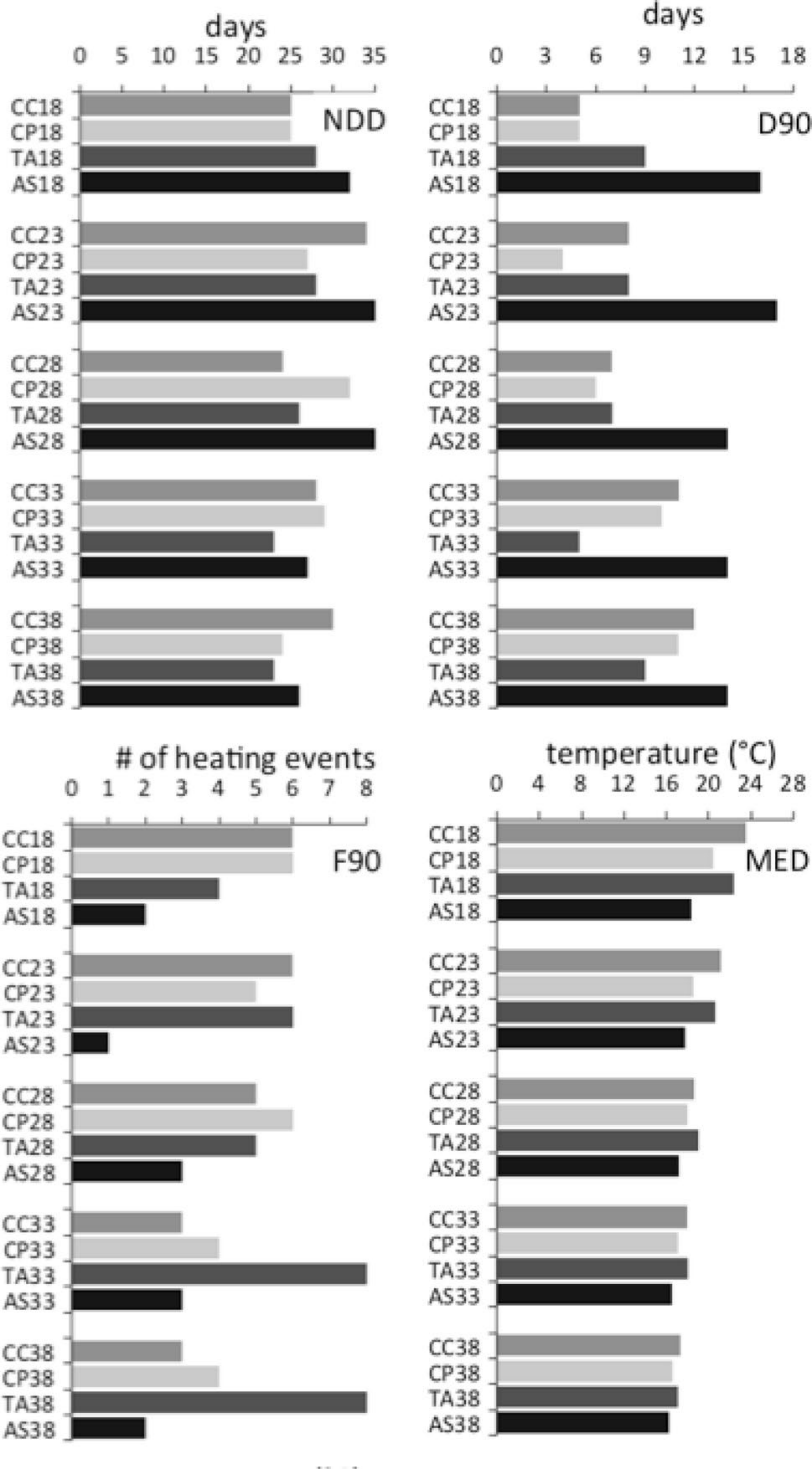
Variability of the explanatory variables considered (D90, MED, NDD and F90) at each site (AS, CP, TV, and CC) and depth (18 m, 23 m, 28 m, 33 m and 38 m).

A more accurate description of such relevant heterogeneity in thermal regimes among depths and sites was entrusted to the four heating descriptors (D90, MED, NDD and F90, the explanatory variables) selected by the models (from a set of 26, Table 1). Therefore aspects of the heating events were quantified by each descriptor: (1) duration by D90, the maximum duration (in days) of events warmer than the 90th percentile temperature; (2) heat intensity by MED, the median temperature and (3) temperature variability by NDD, the number of daily ΔT larger than the mean daily ΔT, and by F90 the number of heating events larger in ΔT than the 90th percentile ΔT (Table 1 and Fig. 3). Consequently, AS is clearly ranked the coldest, while CC and TA are the hottest sites (with CP in between), for the consistency of MED patterns among depths. However, in terms of duration of heating events AS is the one with the longest D90 consistently across depths, while at CC D90 is quite variable and increases with depth; at TA and CP large variations among depths were estimated, and not following a depth gradient. Moreover, NDD and F90 have measured different aspects of temperature variability: AS had the highest NDD and the lowest F90, TA had increasing F90 with depth, but homogeneous and low NDD, and CC had a clear pattern only for F90 which decreased with depth.

**Table 1:** List of all temperature descriptors tested as explanatory variables for the duration of heating events, temperature intensity and variability. In bold are the descriptors selected by the models.

Descriptor type	TAG	Temperature descriptors
**Duration**	**D90**	**Max duration (days) > 90th percentile**
Duration	D95	Max duration (days) > 95th percentile
Duration	D27	Max duration (days) of heat event > 27° C
Duration	D26	Max duration (days) of heat event > 26° C
Duration	D25	Max duration (days) of heat event > 25° C
Duration	D24	Max duration (days) of heat event > 24° C
Duration	D23	Max duration (days) of heat event > 23° C
Intensity	MAXT	Max T
Intensity	AT	Average T
Intensity	MINT	Min T
**Intensity**	**MED**	**Median T**
Intensity	S95	Sum of days > 95th percentile
Intensity	S90	Sum of days > 90th percentile
Intensity	S27	Sum of days > 27° C
Intensity	S26	Sum of days > 26° C
Intensity	S25	Sum of days > 25° C
Intensity	S24	Sum of days > 24° C
Intensity	S23	Sum of days > 23° C
**Variability**	**F90**	**# of heating events > 90th percentile**
Variability	F95	# of heating events > 95th percentile
Variability	F24	# of heating events > 24° C
**Variability**	**NDD**	**# daily ΔT > mean daily ΔT**
Variability	NHS	# of heating shifts (> 4° ΔT) in two days ^∧^
Variability	SD	Standard deviation
Variability	LDH	Largest daily heating (ΔT, in °C)
Variability	LTH	Largest heating (ΔT, in °C) in two days^∨^

Furthermore, based on the SST climatology of the four sites, May–October 2019 SST data (Fig. 2) were used to detect and characterize the MHWs during the study^15,48^: three MHWs were found in AS and CC, four in CP and two in TA. MHWs differed in intensity and duration: some strong waves have occurred at all the sites, but they were not directly traceable in the subtidal temperature (Table S1 and Fig. S2, supplementary 1).

### Coralligenous structure-heating descriptors models

A total of 42 taxa/morphological groups composing the coralligenous reef community were identified at several taxonomic levels (18 macroalgae, 7 anthozoans, 7 bryozoans, 5 sponges, 2 polychaete, 2 tunicates and 1 hydrozoan, Table 2) and used to calculate the community richness and evenness.

**Table 2:** Presence (+) or absence (−) of coralligenous taxa/morphological groups at all the sites (AS, CP, TA and CC) and depth (18, 23, 28, 33 and 38 m).

Taxa/morphological groups	Cat	AS					CP					TA					CC				
		18	23	28	33	38	18	23	28	33	38	18	23	28	33	38	18	23	28	33	38
Articulated Coralline Algae	C	+	+	+	+	+	+	+	+	+	+	+	+	+	−	+	−	−	−	+	+
*Asparagopsis* sp*.*	R	−	−	−	−	−	+	−	−	−	−	−	−	−	−	−	−	−	−	−	−
*Caulerpa cylindracea*	G	−	−	−	−	−	−	−	−	−	−	−	−	−	−	−	−	−	−	+	+
*Codium* spp*.*	G	+	+	−	+	−	+	+	+	−	−	−	−	−	+	−	−	−	+	+	+
Dictyotales		+	+	+	+	+	+	+	−	+	+	+	+	+	+	+	+	+	+	+	−
Encrusting Coralline Algae	C	+	+	+	+	+	+	+	+	+	+	+	+	+	+	+	+	+	+	+	+
Encrusting Ochrophyta		+	+	+	+	+	−	−	−	+	+	−	−	+	−	+	−	+	+	−	−
Erect flattened Rhodophyta	R	−	+	+	+	+	−	−	−	−	−	−	−	−	+	+	−	+	−	−	+
Erect terete Ochrophyta		+	+	+	+	+	−	−	−	−	−	−	−	−	+	+	−	−	−	−	−
Erect terete Rhodophyta	R	+	+	+	+	+	+	+	+	+	+	+	+	−	+	+	−	−	+	+	+
*Flabellia petiolata*	G	+	+	+	+	+	+	+	+	+	+	+	+	+	+	+	−	+	+	+	+
Fucales		+	+	−	+	+	−	−	−	−	−	−	−	−	−	−	−	−	−	−	−
*Halimeda tuna*	G	+	+	+	+	+	+	+	+	+	+	+	+	+	+	+	−	−	+	−	−
*Palmophyllum crassum*	G	+	+	+	+	+	+	+	+	+	+	+	+	+	+	+	+	+	+	+	+
*Peyssonnelia* sp*.*	R	+	+	+	+	+	+	+	+	+	+	+	+	+	+	+	+	+	+	+	+
*Pseudoclorodesmis furcellata/Bryopsis* sp.	G	−	+	−	+	+	+	+	+	−	+	−	+	−	−	+	−	−	−	+	−
Turf algae	T	+	+	+	+	+	+	+	+	+	+	+	+	+	+	+	+	+	+	+	+
*Valonia* sp*.*	G	−	+	+	+	+	+	+	+	+	+	+	+	+	+	+	+	+	+	+	+
*Aedonella calveti*	B	−	−	+	+	+	−	−	−	+	+	−	+	+	+	+	−	+	+	+	+
Encrusting bryozoans	B	+	+	+	+	+	+	+	+	+	+	+	+	+	+	+	+	+	+	+	+
*Myriapora truncata*	B	+	+	+	+	+	+	+	+	+	+	−	+	+	+	+	−	−	−	+	+
*Pentapora fascialis*	B	+	+	+	+	−	−	−	−	+	+	−	+	+	+	+	−	−	+	+	+
Ramified bryozoans	B	−	+	+	+	+	+	+	+	+	+	+	+	+	+	+	+	+	+	+	+
*Reteporella grimaldii*	B	−	−	+	−	+	+	+	+	+	+	−	−	+	+	+	−	−	+	+	+
*Smittina cervicornis*	B	+	+	+	+	+	−	−	+	+	+	−	+	+	+	+	−	−	+	+	+
Hydrozoans		−	+	+	+	+	−	−	−	−	+	+	+	+	+	+	−	−	+	+	+
*Alcyonum coralloides*		−	−	+	+	+	−	−	−	−	−	−	−	−	+	+	−	−	−	−	+
*Corallium rubrum*		−	−	−	−	−	−	+	+	+	+	−	−	−	−	−	−	−	−	−	−
*Eunicella cavolini*		−	−	+	+	+	−	−	+	+	+	−	+	+	+	+	−	−	+	+	+
*Paramuricea clavata*		−	−	−	−	−	−	−	−	−	−	−	−	+	+	+	−	−	−	+	+
*Parazoanthus axinellae*		+	+	+	+	+	−	−	+	+	+	+	+	+	+	+	+	+	+	+	+
Azooxantellate individual scleractinians		+	+	+	+	+	−	+	+	+	−	+	−	+	+	+	−	−	+	+	+
Zooxantellate scleractinians		+	+	+	+	+	−	+	+	+	−	+	−	+	+	+	−	−	+	+	+
Large serpulids		−	+	+	+	+	+	+	+	+	+	+	+	+	+	+	+	+	+	+	+
*Salmacina-Filograna* complex		−	+	−	−	−	−	+	−	+	+	+	+	+	+	+	−	+	+	+	+
Arborescens/massive sponges	S	+	+	+	+	+	−	+	+	+	+	+	+	+	+	+	−	−	+	+	+
Bushy sponges	S	+	+	+	+		−	+	+	+	+	+	+	+	−	+	−	−	−	+	+
Encrusting sponges	S	+	+	+	+	+	+	+	+	+	+	+	+	+	+	+	+	+	+	+	+
Perforating sponges	S	−	−	−	−	−	−	−	−	+	+	−	−	+	−	−	−	−	+	−	+
Prostrate sponges	S	−	−	−	+	−	+	+	+	+	+	+	+	−	+	−	−	+	+	+	+
Erect ascidians		−	+	+	+	+	−	+	−	+	+	+	+	+	+	+	+	+	+	−	+
Encrusting ascidians		−	−	−	−	−	+	−	−	−	−	−	−	−	+	−	−	−	−	−	−

Taxa categories (Cat.) of GAM models are indicated by the letters *B* bryozoans, *C* encrusting coralline algae, *G* green algae, *S* sponges, *R* red algae and *T* turf algae.

**Figure 4:**
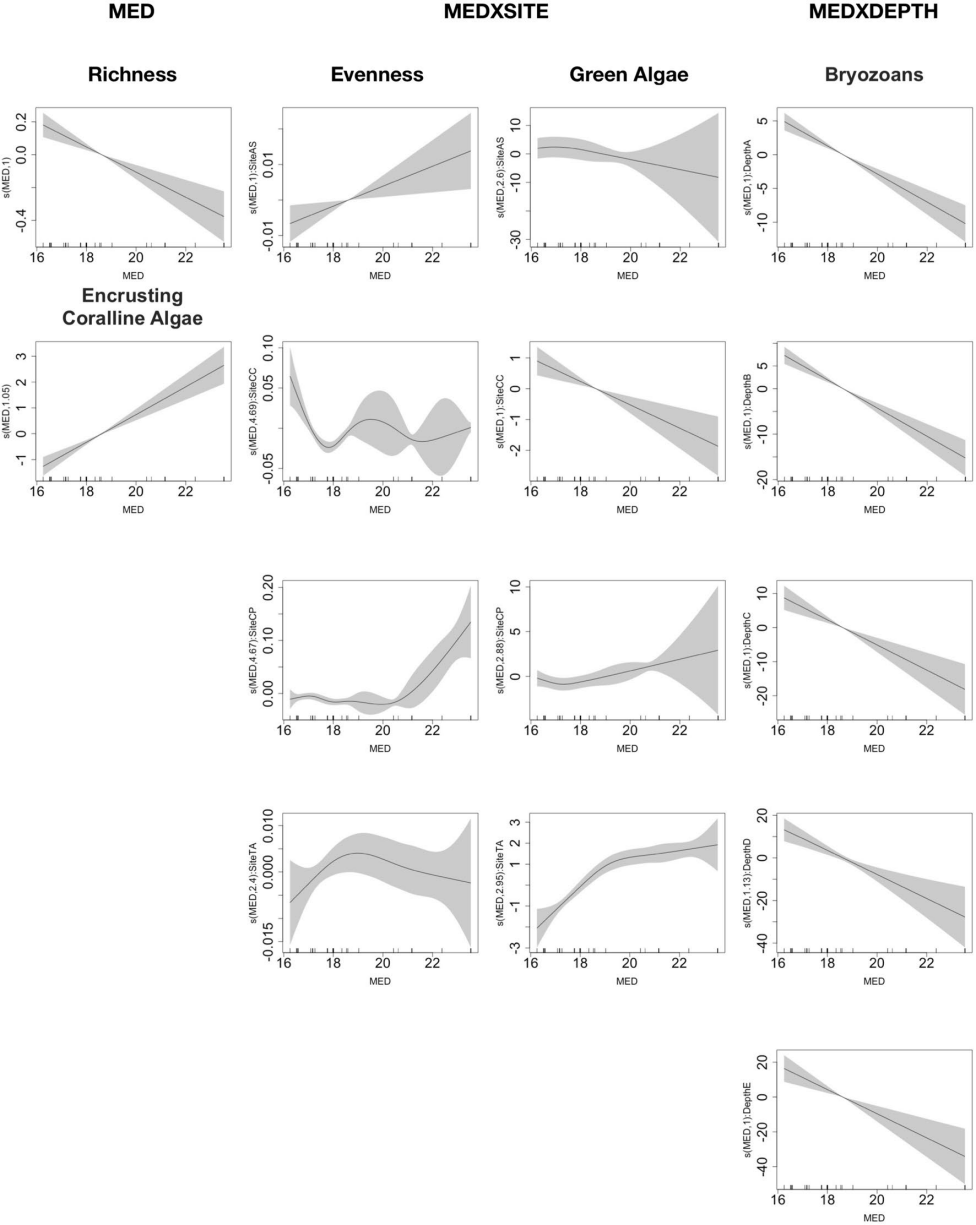
Summary of GAM results showing only the significant explanatory variables of the models: MED both, as a main factor and MED x SITE (AS, CP, TV, and CC), and x DEPTH (18 m = A, 23 m = B, 28 m = C, 33 m = D and 38 m = E). Th.e y-axis represents the additive predictor by GAM, which is a smoothed function for MED and for the interaction. The solid line is the smoother and the dotted lines are 95% point-wise confidence bands.

**Figure 5:**
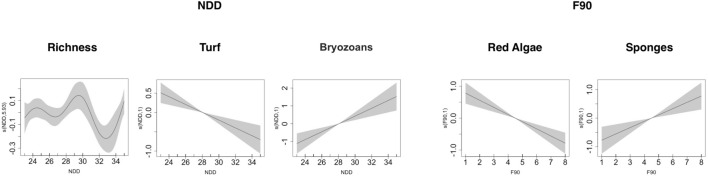
Summary of GAM results showing only the significant explanatory variables of the models: NDD and F90. The y-axis represents the additive predictor by GAM, which is a smoothed function for NDD and for the interaction. The solid line is the smoother and the dotted lines are 95% point-wise confidence bands.

The models have highlighted the significant influence of the site on the community (in terms of taxa richness and evenness) and on all the categories of taxa considered, while depth only affected a few of the response variables: encrusting coralline algae, red algae, turf algae, sponges and bryozoans (Table 3 and Fig. S3 supplementary 1). However, the most interesting result was about the influence of the selected explanatory variables (D90, MED, F90 and NDD), alone or in interaction with site and depth on the coralligenous community structure (richness and evenness) and every single conspicuous category (supplementary 2). Surprisingly, all the coralligenous response variables were influenced by some of the heating descriptors (Table 3). In fact, both intensity and variability of heating events influenced the number of taxa of the community, as richness was negatively influenced by the MED larger than 18.5 °C, while it had a non-linear response to NDD (Figs. 4, 5 and Table 3. Conversely, the community evenness was only affected by MED intensity depending on the site, except for TA (Table 3 and Fig. 4).

**Table 3:** Summary of the significant GAMs results showing only the significant explanatory selected variables: site, depth and the temperature descriptors for the heating duration, temperature intensity and variability. N = 400. Dev = the deviance explained by the models. “X” = interaction.

Response variable			Heating duration	Heating intensity	Heating variability		Dev
**Community**							
Richness	Site		D90	MED	NDD		58.2%
Evenness	Site			MEDXSite			25.5%
**Category**							
Green algae	Site			MEDXSite			65.4%
Bryozoans	Site	Depth	D90XSite	MEDXDepth	NDD		38.7%
Turf algae	Site	Depth	D90XDepth		NDD		34.8%
Red algae	Site	Depth	D90XSite			F90	29.7%
Sponges	Site	Depth	D90XSite			F90	29.0%
Encr Coralline algae	Site	Depth	D90XDepth	MED			26.7%

**Figure 6:**
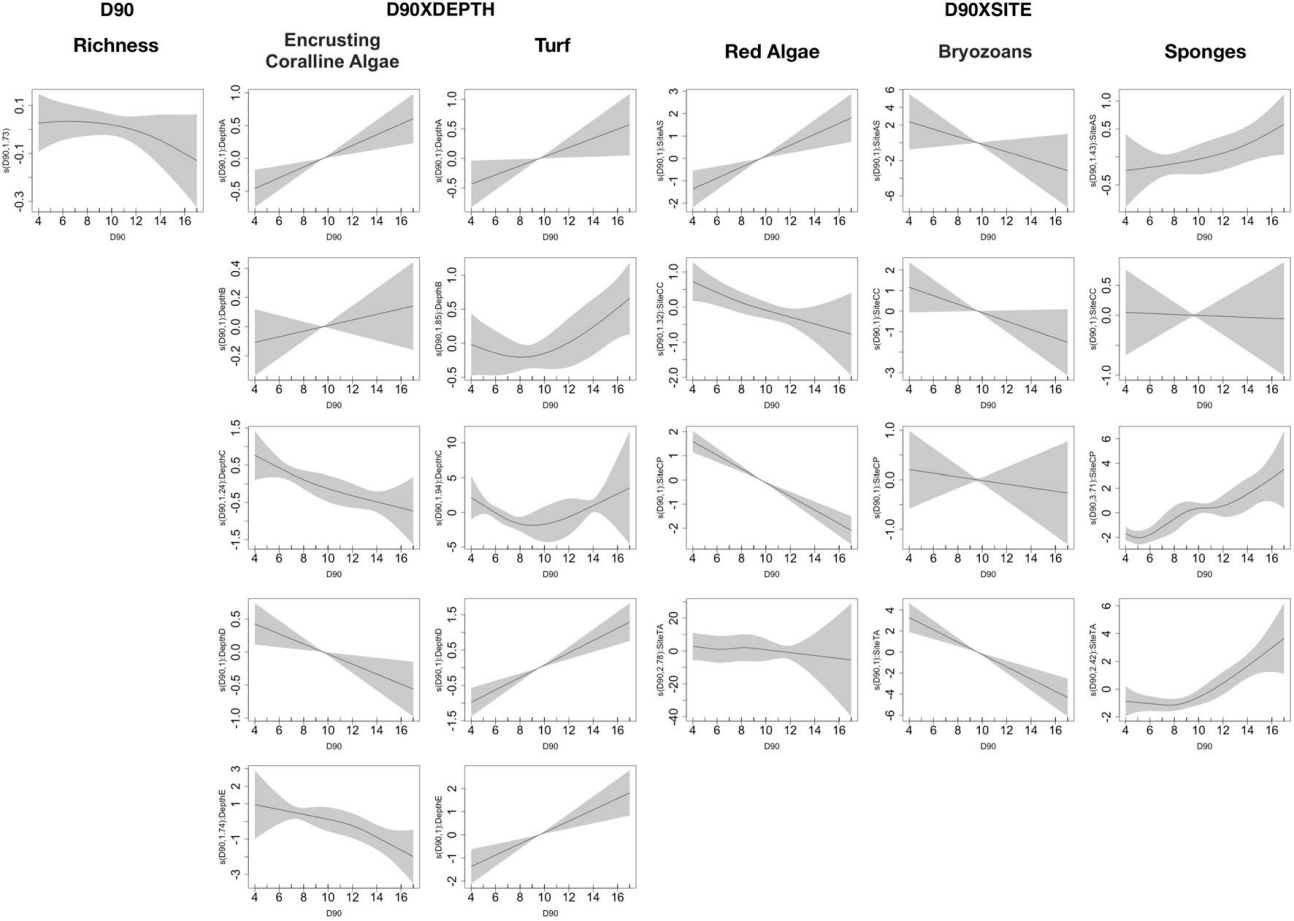
Summary of GAM results showing only the significant explanatory variables of the models: D90 both as main factor and D90 × DEPTH (18 m = A, 23 m = B, 28 m = C, 33 m = D and 38 m = E), and × SITE (AS, CP, TV, and CC). The y-axis represents the additive predictor by GAM, which is a smoothed function for D90 and for the interaction. The solid line is the smoother and the dotted lines are 95% point-wise confidence bands.

Particularly, the duration of heating events (D90, Table S2 in supplementary 1) has influenced all the categories of taxa (except for the green algae), though interactively with depth or site (Fig. 6). D90 had a positive influence on the abundance of turf algae at all depths (with 10 being the most common threshold) and encrusting coralline algae only at 18 m, while deeper the influence had an opposite direction, with the same threshold (10). Furthermore, the site also changed the type of D90 influence on some categories (red algae were negatively influenced at CC and CP, but positively at AS, while sponges were positively influenced at both AS and CP and bryozoans negatively at TA), although exceeding 10 D90 always seemed to make a difference.

The 18.5 °C MED intensity of heating events seemed to be an important threshold as encrusting coralline algae and bryozoans received a positive and negatively effect, respectively, for temperature larger than 18.5 °C. Furthermore, the type of effect on the green algae depended on the site but 18.5 °C remained the threshold (Table 3 and Fig. 4). Moreover, variability in heating events did have an influence on some categories: turf and red algae were negatively affected by NDD larger than 28 and F90 larger than 4.5, while the same descriptors had a positive influence on bryozoans and sponges again for NND larger than 28 and F90 larger than 4.5 (Fig. 5 and Table 3).

## Discussion

In a temperate location like Sardinia, the dynamics of subtidal temperature changes relevantly among sites and depths during the water stratification period. The whole study refers to the heating events that have occurred in the water column independently of the surface MHWs, whose lag of influence in time and space on the deep water temperature has likely been driven by several hydrodynamic mechanisms^57^: at each study site, satellite-derived SST was correlated with logger measures down to 38 m deep (Table S3 supplementary 1), but this did not allow accurate predictions on the thermal subtidal environment^58,59^. Therefore, having detected the MHWs during the study period was useful to characterize the surface conditions, although their direct effects on the deeper thermal environment still remain to be deeply explored.

The objective limitation of not having long time-series of subtidal temperature data has forced to find proxies of heating events, resembling the MHWs metrics^60,61^. All the heating descriptors here selected, D90, MED, NDD and F90, influenced several coralligenous response variables either of the community or the main categories, so that they all might be considered useful predictors for climate change investigations on the coralligenous reef. Accordingly to the correlations among temperature descriptors highlighted during data exploration, the predicting value of those selected can be extended to the correlated descriptors (see below data analysis). The possibility of exploiting the use of such descriptors of heating events for other subtidal efforts should be investigated, in addition to MHWs metrics for the surface^15,48^. Currently, the response of other subtidal community/species to the thermal environment is being examined based on satellite-derived SST, if the systems investigated are very shallow^62^. Alternatively, studies for deeper systems have relied on in situ loggers measures where, for very specific locations, temperature data have started to be collected long before the study was really designed and where researchers could formulate the hypotheses only a posteriori^60–65^. However, estimating subtidal temperature anomalies based on the subtidal climatology by long-time series of field temperature data has to be supported, although it will concern a limited number of localities: the recent promotion of research projects or temperature monitoring programs (i.e. TMED for the Mediterranean, http: //t-mednet) that have extensively implemented logger data acquisition will be fundamental to gain information in wide areas and will enable detecting the role of sea water warming on relevant ecological patterns (i.e. mass mortalities) by correlative analyses^66^.

Overall, the arbitrary choices made to define the D90, MED, NDD and F90 descriptors were striking, as far as there have been many significant associations with the coralligenous response variables selected. Among the whole portfolio of the coralligenous taxa/morphological groups initially considered (before pooling data) in the analyses, none could be considered due to the negligible sample size for most of them and results could be obtained only from models run on conspicuous groups of taxa (obtained by data pooling). This represents a relevant bias of the methodology, especially for this habitat since the coralligenous community is importantly structured by a multitude of taxa with a natural very low area of occupancy^67^ and this approach does not allow deriving any prediction for uncospicuous taxa. This aspect involves even the cnidarian species, iconic taxa to the habitat, that have been repeatedly affected in the Mediterranean by relevant mortality events during summer–autumn heat waves (i.e*.* in 1985, 1999, 2003 and 2008)^36,37,46,68^ and whose disappearance may cause shifts in the community composition, favoring filamentous algae^69^. Change in the structure of coralligenous reefs with loss of species (lower richness and diversity) is a wide described phenomenon^61^. Although for the study sites there is lack of historical complete quantitative data, information about gorgonians mortalities gained for TA site^36,37,40^ and CC [FP, personal observation], induce inferring that even the structure of the communities currently under focus is the product of pressing climate alteration.

Nevertheless, the approach used in this study can only lead formulating predictions on large categories of taxa. In general, the expectation about the coralligenous community change to heating events, based on the overall vulnerability to warming treatments^38,39,45,60^, concerns the reduction of sea fan and encrusting sponge species abundance and the consequent increase of algae such as turfs or fast growing weeds^61^. In this study, although with the limit of the taxonomic resolution, some unequivocal predictions of the effects of heating events on the coralligenous community could be made, as there was a consistence response across sites and depths of associations (descriptor-coralligenous): these include the high predictive power of the median temperature for the taxa richness, for the encrusting coralline algae and bryozoans. Based on these, increases in temperature intensity would drive to a lower number of taxa and, among the categories composing the community, to an increase and decrease of encrusting coralline algae and bryozoans, respectively. This prediction is in accordance with the recent investigations on the whole community^61^ and on bryozoans^70^, although for these latter temperature tolerance seems species-dependent^44^ and varies among areas^71^. Conversely, experimental evidence on the response of encrusting coralline algae to heat is badly needed^72^. Moreover, in the projection of longer lasting heating events, turf algae will increase the spread, as they were positively associated to D90, evidencing that the duration longer than 10 days is likely the key aspect of heating events responsible for the increase in turfs on the coralligenous reefs^61^.

Measures of heating variability, NDD and F90, have provided some further important insights, as they were good predictors for the decrease in turf (NDD over 28 daily temperature shifts larger than the mean daily shift) and red algae (F90 over 4.5 heating events larger than the 90th percentile), and increase in sponges (F90 larger than 4.5) and bryozoans (NDD larger than 28). This result may be seen in apparent contrast with expectations, but the most likely interpretation is that large values of these two descriptors correspond to breaks to the trajectories of change driven by increases in intensity and duration of heating events, stressing the need of not neglecting heat variability in studies of community responses to sea water warming. At this regards it seems important to highlight that NDD and F90 do not estimate the frequency of heating events, an aspect of heating events that was not taken into consideration in this study.

Certainly, important thresholds for whatever type of effect (negative or positive) on the coralligenous community were found for all the temperature descriptors: 18.5 °C for the median temperature, 28 for NDD, 4.5 for F90 and 10 for D90. Although related to the specific study period that does not include the cold months, all these thresholds together provide evidence for formulating predictions on the coralligenous reef community on the basis of the heating events. In fact, independently of their absolute values, they seem edges highly consistent across the response variables of the community investigated, suggesting that specific temperature intensity, variability and duration of heating events affect the coralligenous community, both as a whole and its conspicuous taxa.

Overall, discriminating the effect and quantifying the specific magnitude of influence of the heating events, whether the duration, intensity or variability represents one of the main results of the approach used. The other output regards the context-dependence of several predictions, either on the community or the categories of taxa, as the type of associations (negative or positive) changed among sites or depths. However, the categories of taxa are composed of numbered species whose contribution in abundance changes depending on the sitexdepth context and, because vulnerability to the heating may be species specific^44,45^ and often depends on local adaptation^73^, the interpretation of unconsistent patterns could only be very oddly and speculative, until the relevancy of each single species is assessed.

The present study uses the approach of relating different coralligenous communities to their thermal environment in order to contribute drawing the trajectories of change of the coralligenous community due to future warming scenarios. The innovative approach consists in providing some descriptors of heating events and the thresholds to which for this community would change. The same approach could be similarly applied to investigate the responses of any other subtidal community/species to climate change. The need of disentangling the effects due to the intensity, duration and variability of the heating events is stressed, as well as the importance of gaining spatially and temporally wide databases, both of the thermal environment and the biota. We hope the present contribution my also assist the implementing of persistent monitoring nets of subtidal habitats.

## Methods

### Thermal environment

The thermal environment was estimated by deploying in situ high-resolution (hourly records) temperature loggers (HOBO Pendant Temp/Light MX2202) at five depths (18 m, 23 m, 28 m, 33 m and 38 m) on the coralligenous reef of each site. The hobos were anchored by scuba divers to the substratum using steel nails where the coralligenous layer was sufficiently thick, otherwise a two-component epoxy underwater filler. The data logger recorded from the 4th of May until the 16th of October 2019, for a total of 166 days. The data obtained gave information on the variability in water stratification in the four study sites. Furthermore, 30-years satellite-derived SST data were obtained (AVHRR, https://apdrc.soest.hawaii.edu) and used to detect the 2019 MHWs at each site and to explore the correlative pattern with subtidal water temperature.

### Biota data collection

Samplings of the coralligenous community structure were done in May 2019 on vertical rocky walls using a non-destructive photographic method (underwater camera Lumix TZ30 with lighting achieved using two electronic strobes fitted with diffusers). At each site, two areas (about 100 m distant) were selected and at each depth (18 m, 23 m, 28 m, 33 m, 38 m) ten photographic samples of 0.2 m^2^ of vertical surface were taken^74^. Organisms easily detectable from the photograph samples were identified to the lowest possible taxonomic level, while those not easily recognized were identified according to morphological groups^75^. In order to run reliable models, the cover of several unconspicuous taxa/groups was pooled in six categories (encrusting coralline algae, red algae, green algae, turf algae, sponges and bryozoans), based on the sample size and affinities (Table 2). Red and green algae categories were mainly composed of Peyssonneliaceae and Udoteaceae, respectively, while turfs were algae smaller than 1 cm.

The percent cover of each taxon/morphological group was estimated in each picture by ImageJ software (https://imagej.nih.gov/ij/). Richness of taxa/morphological groups and evenness index were calculated from the data acquired.

### Temperature descriptors

A total of 26 temperature descriptors for all sitexdepth conditions were considered to estimating the duration of heating events, their intensity and temperature variability (Table 1). Overall, arbitrary thresholds were set choosing (1) temperature intensity for estimating S23-S27 (the number of days of daily temperature) and D23-D27 (the maximum heating duration) over 23–27 °C, respectively, (2) 4 °C as shift intensity for NHS and (3) 2 days of time for shifts in LTH. However, to estimate differences among sitexdepth conditions in terms of temperature anomalies, the daily temperature data were also explored to identify the number of days of maximum duration of a heating event (D90 and D95), the number of days of high temperature (S90 and S95) and the number of heating events (F90 and F95) respect to the 90th and 95th percentile, calculated on the temperature data collected during 2019. The temperature variability was measured by other several descriptors: NHS, the number of times that heating shifts occurred in two days in a row were larger than 4 °C; LTH, the largest heating event (ΔT, in °C) occurred in two days in a row; NDD, the number of daily shifts larger than the daily mean shift; LDH the largest daily heating (in °C, Table 1).

### Data analysis

Data exploration was carried out following^76^. Outliers were inspected with Cleveland dot-plots and normality with histograms and Q–Q plots. Collinearity between continuous explanatory variables was inspected with pair-plots, and variance inflation factors (VIFs) were calculated. Several significant correlations were found among covariates: (1) D95, D27, D26, D25, D24, D23 were all correlated with D90; (2) F95 and F24 were correlated with F90; (3) NHS and LDH were correlated with LTH; (4) MAXT, AT, MINT, SD, S90, S95, S27, S26, S25, S24, S23 were all correlated with MED. Thus, only the five covariates MED, D90, F90, NDD, and LTH (VIFs < 2) were considered in the analyses even though the results obtained for each of them can extended to all the correlated descriptors. Conditional scatterplots were used to investigate the potential relationship between the explanatory variables: (i) continuous variables: MED, D90, F90, NDD, and LTH; (ii) Site (categorical with four levels: AS, CC, TA, CP; (iii) Depth (categorical with five levels: A = 18 m, B = 23 m, C = 28 m, D = 33 m, E = 38 m) and the interactions between covariates and factors with the coralligenous response variables.

Since data exploration indicated non-linear relationships between each response variable and the explanatory variables, generalized additive models (GAMs) were run to correlate separately the different response variables with the explanatory variables, allowing detection of the eventual effects of Site and Depth on the continuous variables. GAMs are nonparametric extensions of linear regression models that allow the evaluation of highly non-linear relationships between explanatory and response variables thanks to the use of smooth functions^77^. To avoid over-dispersion, a negative binomial distribution was applied to all the response variables, with the exception of S (Poisson distribution) and evenness (gaussian distribution). The choice of the best fitting explanatory variables used in the final model was undertaken using AIC (Akaike information criterion), following a forward selection approach^76^. Models validation were run calculating and plotting the Pearson residuals against (i) the fitted values, (ii) each explanatory variable in the model, (iii) each explanatory variable not in the model^76^ (supplementary 2).

## Supplementary information

Supplementary file1
