# Supplementary material for: The constraint of ignoring the subtidal water climatology in evaluating the changes of coralligenous reefs due to heating events
Source: Sci Rep. 2020 Oct 15;10:17332. doi: 10.1038/s41598-020-74249-9 (PMC7562739; doi:10.1038/s41598-020-74249-9)
Supplement: Supplementary file 1 — Supplementary file1 [file 41598_2020_74249_MOESM1_ESM.docx]

**THE CONSTRAINT OF IGNORING THE SUBTIDAL WATER CLIMATOLOGY IN EVALUATING THE CHANGES OF CORALLIGENOUS REEFS DUE TO HEATING EVENTS**

Giulia Ceccherelli^1*^, Federico Pinna^1^, Arianna Pansini^1^, Luigi Piazzi^1^, Gabriella La Manna^2^

**^1^**Dipartimento di Chimica e Farmacia, Università di Sassari, via Piandanna 4 07100 Sassari Italy.

**^2^**MareTerra Onlus – Environmental Research and Conservation, 07041 Alghero SS (Italy).

*Correspondence and request for materials should be addressed to G.C. (cecche@uniss.it)

**supplementarY materialS 1: SST analySIS**

Table S1 List of all MHWs detected from the 4^th^ of May to the 16^th^ of October 2019 at the sites (AS, TA, CC and CP). For each of them the duration and the intensity category (moderate, strong, severe or extreme) was calculated based on Hobday et al 2018. The letters a, b, c and d refer to the MHW of each site, counting from May through October in sequence. Category I and II refer to the MHW intensity. *P* is the percentage of time spent in each of the MHW categories over the duration of the event.

| **site** | **MHW** | **category** | **peak date** | **Duration**  **(days)** | **Max Intensity**  **(°C)** | ***P* moderate** | ***P* strong** | ***P* severe** | ***P* extreme** |
| --- | --- | --- | --- | --- | --- | --- | --- | --- | --- |
| CC | a | II | 06-Jul | 11 | 0.96 | 91 | 9 |  |  |
|  | b | I | 05-Sept | 5 | 0.50 | 100 |  |  |  |
|  | c | II | 26-Sept | 12 | 1.03 | 92 | 8 |  |  |
| TA | a | II | 06-Jul | 12 | 1.51 | 83 | 17 |  |  |
|  | b | I | 27-Aug | 6 | 0.88 | 100 |  |  |  |
| CP | a | II | 05-Jul | 13 | 1.78 | 85 | 15 |  |  |
|  | b | II | 31-Aug | 10 | 0.81 | 90 | 10 |  |  |
|  | c | I | 28-Sept | 8 | 0.45 | 100 |  |  |  |
|  | d | I | 13-Oct | 5 | 0.38 | 100 |  |  |  |
| AS | a | II | 08-Jul | 14 | 1.92 | 86 | 14 |  |  |
|  | b | II | 31-Aug | 14 | 0.89 | 93 | 7 |  |  |
|  | c | I | 25-Sept | 11 | 0.66 | 100 |  |  |  |

Table S2 Temperature (°C) of the 90^th^ percentile used to calculate D90 for each site x depth combination.

|  | **Site** | | | | | |
| --- | --- | --- | --- | --- | --- | --- |
|  | | **CC** | **TA** | **CP** | **AS** |  |
| **Depth**  18m | | 26.14 | 24.97 | 25.40 | 22.90 |  |
| 23m | | 25.30 | 24.23 | 23.66 | 22.29 |  |
| 28m | | 23.43 | 23.39 | 23.22 | 20.90 |  |
| 33m | | 22.34 | 22.37 | 22.03 | 18.89 |  |
| 38m | | 20.38 | 22.38 | 19.97 | 18.16 |  |

Table S3 Correlations (all p<0.05) between SST (by satellite) and deep temperature (by loggers) in N=166 days.

|  | **Site** | | | | | |
| --- | --- | --- | --- | --- | --- | --- |
|  | | **CC** | **TA** | **CP** | **AS** |  |
| **Depth**  18m | | r =0.83 | r =0.84 | r =0.71 | r =0.65 |  |
| 23m | | r =0.73 | r =0.72 | r =0.57 | r =0.61 |  |
| 28m | | r =0.56 | r =0.61 | r =0.51 | r =0.58 |  |
| 33m | | r =0.48 | r =0.52 | r =0.43 | r =0.57 |  |
| 38m | | r =0.42 | r =0.40 | r =0.40 | r =0.58 |  |


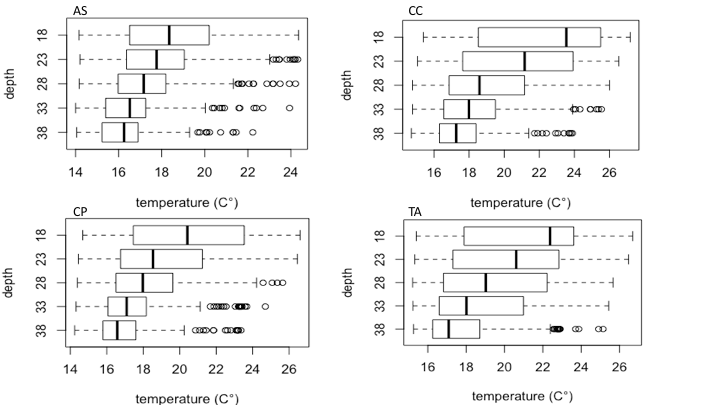


Figure S1. Variability of the temperature as function of the site (AS, CP, TV, and CC) and depth (18m, 23m, 28m, 33m and 38m).


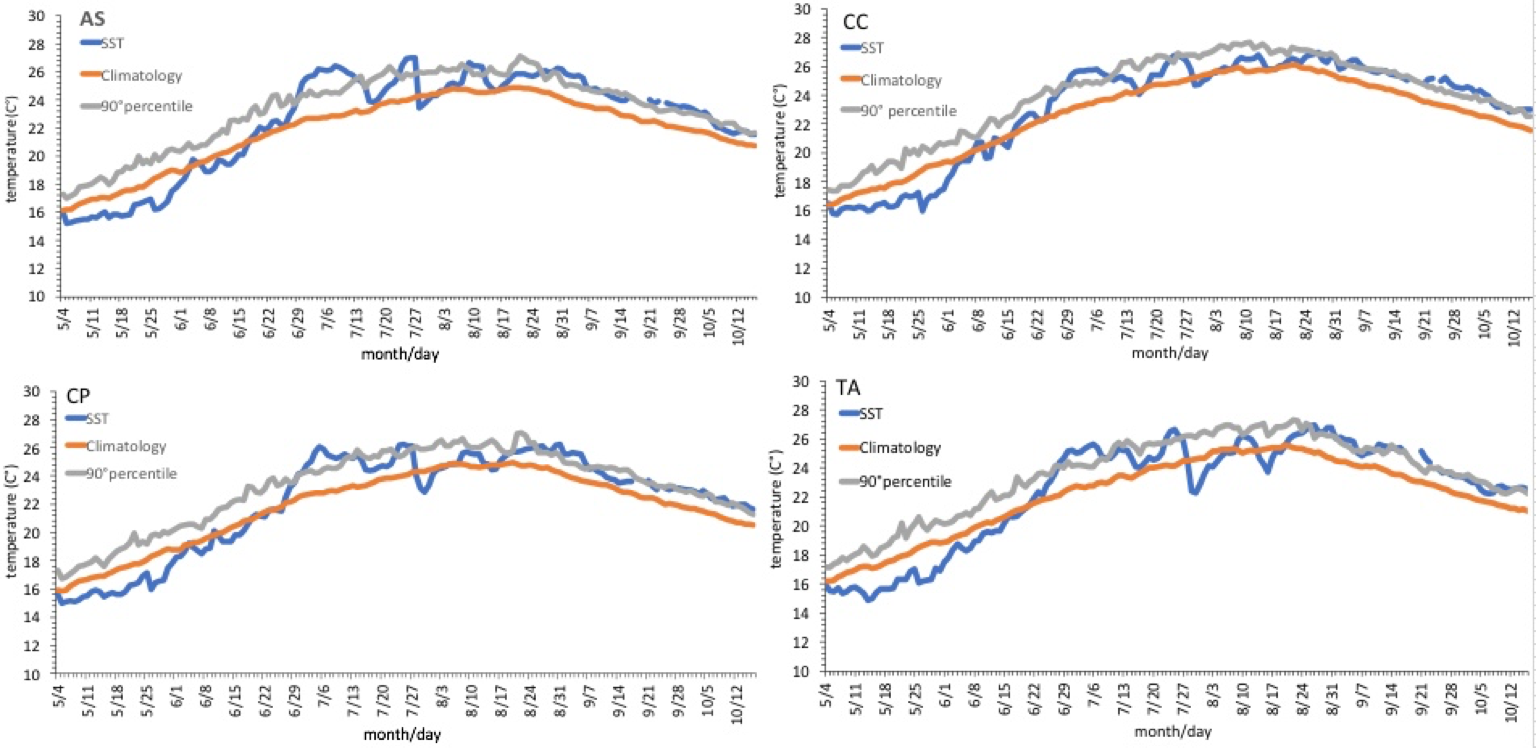


Figure S2. Daily temperature from May 4 to October 16 2019 at the four sites (AS, CP, TV, and CC): in blue SST (from satellites) of 2019, in orange the SST climatology and in grey the SST 90^th^ percentile.


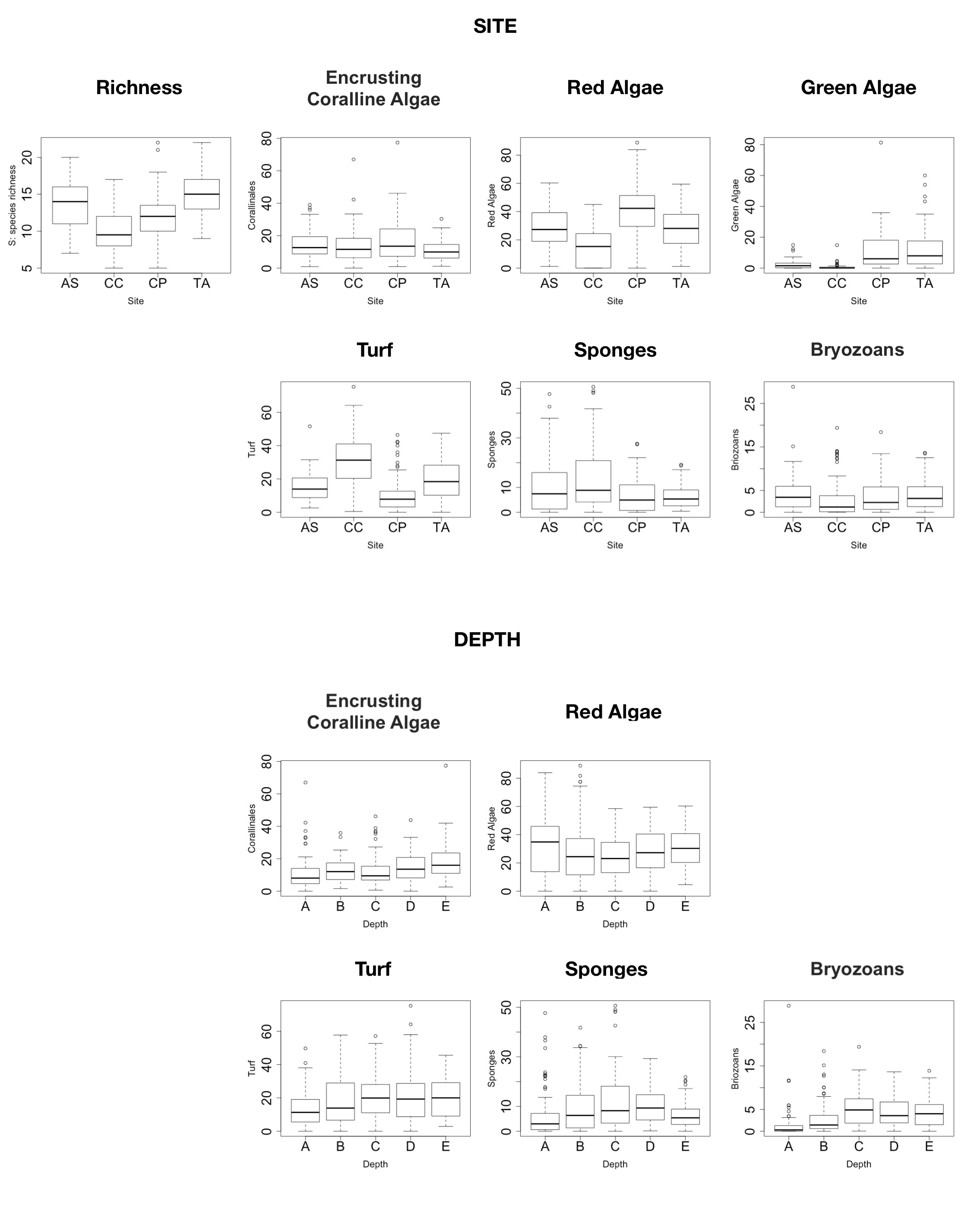


Figure S3. Variability of the response variables as function of the site (AS, CP, TV, and CC) and depth (18m, 23m, 28m, 33m and 38m).

**supplementarY materialS 2: MODEL RESULTS**

**Results: RICHNESS**

Table S4. Statistical details for smooth and parametric terms of the final GAM models for richness.

| **Richness** | | | | |
| --- | --- | --- | --- | --- |
| **Smooth term** | **df** | **Chi-square** | **P-value** |  |
| s(MED) | 1 | 24.047 | **< 0.0001** |  |
| s(D90) | 2.092 | 1.982 | 0.424132 |  |
| s(NDD) | 6.964 | 29.505 | **0.000125** |  |
| **Parametric terms** | **Estim.** | **Std. Er.** | **Z-value** | **P-value** |
| Intercept | 2.57852 | 0.07595 | 33.95 | **< 0.0001** |
| factor(Site)CC | -0.25896 | 0.10168 | -2.547 | **0.0109** |
| factor(Site)CP | -0.1813 | 0.10558 | -1.717 | 0.086 |
| factor(Site)TA | 0.15334 | 0.10875 | 1.41 | 0.1586 |
| **Deviance explained: 58.2% n= 400** | | | | |

**Results: Evenness**

Table S5. Statistical details for smooth and parametric terms of the final GAM models for Evenness

| **Evenness** | | | | |
| --- | --- | --- | --- | --- |
| **Smooth term** | **df** | **F** | **P-value** |  |
| s(MED):SiteAS | 1.000 | 5.474 | **0.0198** |  |
| s(MED):SiteCC | 3.953 | 6.469 | **< 0.0001** |  |
| s(MED):SiteCP | 3.587 | 2.983 | **0.02** |  |
| s(MED):SiteTA | 2.480 | 1.603 | 0.2329 |  |
| **Parametric terms** | **Estim.** | **Std. Er.** | **Z-value** | **P-value** |
| Intercept | 0.966973 | 0.002715 | 356.113 | **< 0.0001** |
| factor(Site)CC | -0.007981 | 0.009253 | -0.863 | 0.389 |
| factor(Site)CP | -0.016361 | 0.012728 | -1.285 | 0.199 |
| factor(Site)TA | -0.002207 | 0.003145 | -0.702 | 0.483 |
| **Deviance explained: 25.6% n= 400** | | | | |

**Results: Corallinales**

Table S6. Statistical details for smooth and parametric terms of the final GAM models for Coralline algae.

| **Corallinales** | | | | |
| --- | --- | --- | --- | --- |
| **Smooth term** | **df** | **Chi-square** | **P-value** |  |
| s(MED) | 1.103 | 59.434 | **< 0.0001** |  |
| s(D90):DepthA | 1.001 | 10.291 | **0.00134** |  |
| s(D90):DepthB | 1.001 | 0.897 | 0.34372 |  |
| s(D90):DepthC | 1.420 | 10.715 | **0.00155** |  |
| s(D90):DepthD | 1.001 | 7.354 | **0.00669** |  |
| s(D90):DepthE | 2.038 | 18.928 | **0.00011** |  |
| **Parametric terms** | **Estim.** | **Std. Er.** | **Z-value** | **P-value** |
| (Intercept) | 2.035 | 0.152 | 13.386 | **< 0.0001** |
| factor(Site)CC | -1.534 | 0.237 | -6.482 | **< 0.0001** |
| factor(Site)CP | -0.596 | 0.194 | -3.071 | **0.00214** |
| factor(Site)TA | -1.854 | 0.256 | -7.251 | **< 0.0001** |
| factor(Depth)B | 1.020 | 0.144 | 7.080 | **< 0.0001** |
| factor(Depth)C | 1.509 | 0.228 | 6.615 | **< 0.0001** |
| factor(Depth)D | 2.295 | 0.287 | 8.008 | **< 0.0001** |
| factor(Depth)E | 3.044 | 0.484 | 6.296 | **< 0.0001** |
| **Deviance explained: 26.7% n= 400** | | | | |

**Results: Green algae**

Table S7. Statistical details for smooth and parametric terms of the final GAM models for Green Algae

| **Green Algae** | | | | |
| --- | --- | --- | --- | --- |
| **Smooth term** | **df** | **Chi-square** | **P-value** |  |
| s(MED):SiteAS | 3.12 | 19.21 | **0.00031** |  |
| s(MED):SiteCC | 1.002 | 15.05 | **0.000105** |  |
| s(MED):SiteCP | 3.349 | 59.79 | **< 0.0001** |  |
| s(MED):SiteTA | 3.409 | 122.31 | **< 0.0001** |  |
| **Parametric terms** | **Estim.** | **Std. Er.** | **Z-value** | **P-value** |
| (Intercept) | -1.1834 | 1.7975 | -0.658 | 0.5103 |
| factor(Site)CC | 0.7531 | 1.8041 | 0.417 | 0.6764 |
| factor(Site)CP | 3.6104 | 1.8296 | 1.973 | **0.0485** |
| factor(Site)TA | 2.6524 | 1.8018 | 1.472 | 0.141 |
| **Deviance explained: 65.4% n= 400** | | | | |

**Results: RED algae**

Table S8. Statistical details for smooth and parametric terms of the final GAM models for Red Algae

| **Red Algae** | | | | |
| --- | --- | --- | --- | --- |
| **Smooth term** | **df** | **Chi-square** | **P-value** |  |
| s(D90):SiteAS | 1.000 | 11.383 | **0.000735** |  |
| s(D90):SiteCC | 1.561 | 8.597 | 0.044318 |  |
| s(D90):SiteCP | 1.000 | 50.974 | **< 0.0001** |  |
| s(D90):SiteTA | 2.953 | 3.430 | 0.387428 |  |
| s(F90) | 1.004 | 22.655 | **< 0.0001** |  |
| **Parametric terms** | **Estim.** | **Std. Er.** | **Z-value** | **P-value** |
| (Intercept) | 1.2867 | 0.4534 | 2.838 | **0.004541** |
| factor(Site)CC | 0.9738 | 0.3941 | 2.471 | **0.013467** |
| factor(Site)CP | 1.606 | 0.3753 | 4.279 | **< 0.0001** |
| factor(Site)TA | 0.5404 | 4.0025 | 0.135 | 0.892605 |
| factor(Depth)B | -0.4482 | 0.1287 | -3.483 | **0.000497** |
| factor(Depth)C | 0.2158 | 0.1331 | 1.621 | 0.104991 |
| factor(Depth)D | 0.5951 | 0.1699 | 3.502 | **0.000462** |
| factor(Depth)E | 0.7615 | 0.1689 | 4.509 | **< 0.0001** |
| **Deviance explained: 29.7% n=400** | | | | |

**Results: TURF**

Table S9. Statistical details for smooth and parametric terms of the final GAM models for Turfs.

| **Turf** | | | | |
| --- | --- | --- | --- | --- |
| **Smooth term** | **df** | **Chi-square** | **P-value** |  |
| s(D90):DepthA | 1.000 | 4.805 | **0.028398** |  |
| s(D90):DepthB | 1.978 | 8.569 | **0.022097** |  |
| s(D90):DepthC | 1.996 | 21.318 | **< 0.0001** |  |
| s(D90):DepthD | 1.000 | 23.336 | **< 0.0001** |  |
| s(D90):DepthE | 1.000 | 13.709 | **0.000214** |  |
| s(NDD) | 1.003 | 14.852 | **0.000118** |  |
| **Parametric terms** | **Estim.** | **Std. Er.** | **Z-value** | **P-value** |
| (Intercept) | 1.8303 | 0.1578 | 11.600 | **< 0.0001** |
| factor(Site)CC | 1.4303 | 0.2108 | 6.787 | **< 0.0001** |
| factor(Site)CP | 0.1005 | 0.2087 | 0.482 | 0.6302 |
| factor(Site)TA | 0.9886 | 0.2186 | 4.523 | **< 0.0001** |
| factor(Depth)B | 0.6208 | 0.1505 | 4.126 | **< 0.0001** |
| factor(Depth)C | 0.9312 | 0.4452 | 2.092 | **0.0365** |
| factor(Depth)D | 0.2488 | 0.1178 | 2.112 | **0.0347** |
| factor(Depth)E | 0.1530 | 0.2041 | -0.750 | 0.4534 |
| **Deviance explained: 34.8% n= 400** | | | | |

**Results: BRYOZOANS**

Table S10. Statistical details for smooth and parametric terms of the final GAM models for Bryozoans.

| **Briozoans** | | | | |
| --- | --- | --- | --- | --- |
| **Smooth term** | **df** | **Chi-square** | **P-value** |  |
| s(MED):DepthA | 1.000 | 56.866 | **< 0.0001** |  |
| s(MED):DepthB | 1.000 | 60.112 | **< 0.0001** |  |
| s(MED):DepthC | 1.000 | 23.901 | **< 0.0001** |  |
| s(MED):DepthD | 1.251 | 16.251 | **< 0.0001** |  |
| s(MED):DepthE | 1.000 | 18.387 | **< 0.0001** |  |
| s(D90):SiteAS | 1.000 | 2.299 | 0.1294 |  |
| s(D90):SiteCC | 1.000 | 3.58 | 0.0585 |  |
| s(D90):SiteCP | 1.001 | 0.265 | 0.6068 |  |
| s(D90):SiteTA | 1.000 | 23.063 | **< 0.0001** |  |
| s(NDD) | 1.000 | 15.205 | **< 0.0001** |  |
| **Parametric terms** | **Estim.** | **Std. Er.** | **Z-value** | **P-value** |
| (Intercept) | 2.666 | 1.7341 | 1.537 | 0.1242 |
| factor(Site)CC | 5.431 | 2.251 | 2.412 | **0.0158** |
| factor(Site)CP | 1.060 | 1.5716 | 0.674 | 0.5000 |
| factor(Site)TA | 5.156 | 2.1002 | 2.455 | **0.0141** |
| factor(Depth)B | -2.205 | 0.4794 | -4.599 | **< 0.0001** |
| factor(Depth)C | -5.856 | 0.989 | -5.921 | **< 0.0001** |
| factor(Depth)D | -10.856 | 2.0505 | -5.294 | **< 0.0001** |
| factor(Depth)E | -15.816 | 3.3712 | -4.691 | **< 0.0001** |
| **Deviance explained: 38.7% n= 400** | | | | |

**Results: SPONGES**

Table S11. Statistical details for smooth and parametric terms of the final GAM models for Sponges.

| **Sponges** | | | | |
| --- | --- | --- | --- | --- |
| **Smooth term** | **df** | **Chi-square** | **P-value** |  |
| s(D90):SiteAS | 1.001 | 4.726 | **0.0293** |  |
| s(D90):SiteCC | 1.003 | 0.028 | 0.8675 |  |
| s(D90):SiteCP | 3.598 | 53.557 | **< 0.0001** |  |
| s(D90):SiteTA | 1.001 | 0.032 | 0.8582 |  |
| s(F90) | 1.001 | 15.565 | **< 0.0001** |  |
| **Parametric terms** | **Estim.** | **Std. Er.** | **Z-value** | **P-value** |
| (Intercept) | 1.51219 | 0.58409 | 2.589 | **0.00963** |
| factor(Site)CC | 0.74953 | 0.51028 | 1.469 | 0.14187 |
| factor(Site)CP | -0.01079 | 1.29332 | -0.008 | 0.99334 |
| factor(Site)TA | -0.37037 | 0.58555 | -0.633 | 0.52706 |
| factor(Depth)B | 0.27842 | 0.17709 | 1.572 | 0.11591 |
| factor(Depth)C | 0.72273 | 0.184 | 3.928 | **< 0.0001** |
| factor(Depth)D | 0.16529 | 0.25432 | 0.65 | 0.51575 |
| factor(Depth)E | -0.17573 | 0.24119 | -0.729 | 0.46625 |
|  |  |  |  |  |
| **Deviance explained: 29% n= 400** | | | | |

**supplementarY materialS 3: MODEL SELECTION AND VALIDATION**

**Richness model selection. “x” = interaction; “s” = smoother. Distribution: Poisson. In bold the model selected. The model order follows an increasing AIC.**

| Model | Df | AIC | ∆AIC | Dev |
| --- | --- | --- | --- | --- |
| **R ~ factor(Site) + s(MED) + s(D90) + s(NDD)** | **12.67** | **1929.71** | **0.00** | **58.2** |
| R ~ factor(Site) + s(MED) + s(NDD) | 10.79 | 1930.24 | 0.54 | 57.2 |
| R ~ factor(Site) + s(MED)+ s(NDD) + s(F90) | 12.40 | 1930.51 | 0.81 | 57.9 |
| R ~ factor(Site) + s(MED) + s(D90) + s(F90) + s(NDD) | 13.56 | 1931.21 | 1.50 | 58.3 |
| R ~ factor(Site) + s(D90) + s(NDD) | 15.41 | 1934.05 | 4.34 | 58.5 |
| R ~ factor(Site) + factor(Depth) + s(MED) + s(D90) + s(F90) + s(NDD) | 16.20 | 1935.40 | 5.70 | 58.6 |
| R ~ factor(Site) + factor(Depth) + s(MED) + s(D90) + s(F90) + s(NDDxDepth) | 17.27 | 1936.36 | 6.66 | 58.9 |
| R ~ factor(Site) + factor(Depth) + s(MED) + s(D90xSite) + s(F90) + s(NDD) | 17.16 | 1936.38 | 6.68 | 58.8 |
| R ~ factor(Site) + factor(Depth) + s(MED) + s(D90) + s(F90) + s(NDD) + s(LTH) | 16.82 | 1936.54 | 6.83 | 58.6 |
| R ~ s(MED) + s(D90) + s(NDD) | 16.78 | 1936.56 | 6.85 | 58.6 |
| R ~ factor(Site) + factor(Depth) + s(MED) + s(D90xDepth) + s(F90) + s(NDD) | 17.90 | 1937.68 | 7.97 | 58.8 |
| R ~ factor(Site) + factor(Depth) + s(MED) + s(D90) + s(F90xDepth) + s(NDD) | 17.94 | 1937.69 | 7.98 | 58.9 |
| R ~ factor(Site) + factor(Depth) + s(MEDxDepth) + s(D90) + s(F90) + s(NDD) | 18.22 | 1937.69 | 7.99 | 59.0 |
| R ~ factor(Site) + factor(Depth) + s(MED) + s(D90) + s(F90) | 16.25 | 1937.90 | 8.20 | 58.0 |
| R ~ factor(Site) + factor(Depth) + s(MED) + s(D90) + s(F90xSite) + s(NDD) | 18.33 | 1938.19 | 8.48 | 58.9 |
| R ~ factor(Site) + factor(Depth) + s(MED) + s(D90) + s(F90) + s(NDDxSite) | 18.29 | 1938.19 | 8.48 | 58.9 |
| R ~ factor(Site) + factor(Depth) + s(MEDxSite) + s(D90) + s(F90) + s(NDD) | 18.37 | 1938.30 | 8.59 | 58.9 |
| R ~ factor(Site) + factor(Depth) + s(MED) + s(D90) | 18.44 | 1938.95 | 9.24 | 58.8 |
| R ~ factor(Site) + factor(Depth) + s(MED) | 15.67 | 1939.06 | 9.35 | 46.3 |
| R ~ factor(Site) + s(MED) + s(D90) | 18.79 | 1939.30 | 9.60 | 58.9 |
| R ~ factor(Site) + factor(Depth) | 8.00 | 1954.05 | 24.34 | 49.9 |
| R ~ factor(Site) | 4.00 | 2035.50 | 105.80 | 27.7 |

**Richness model validation
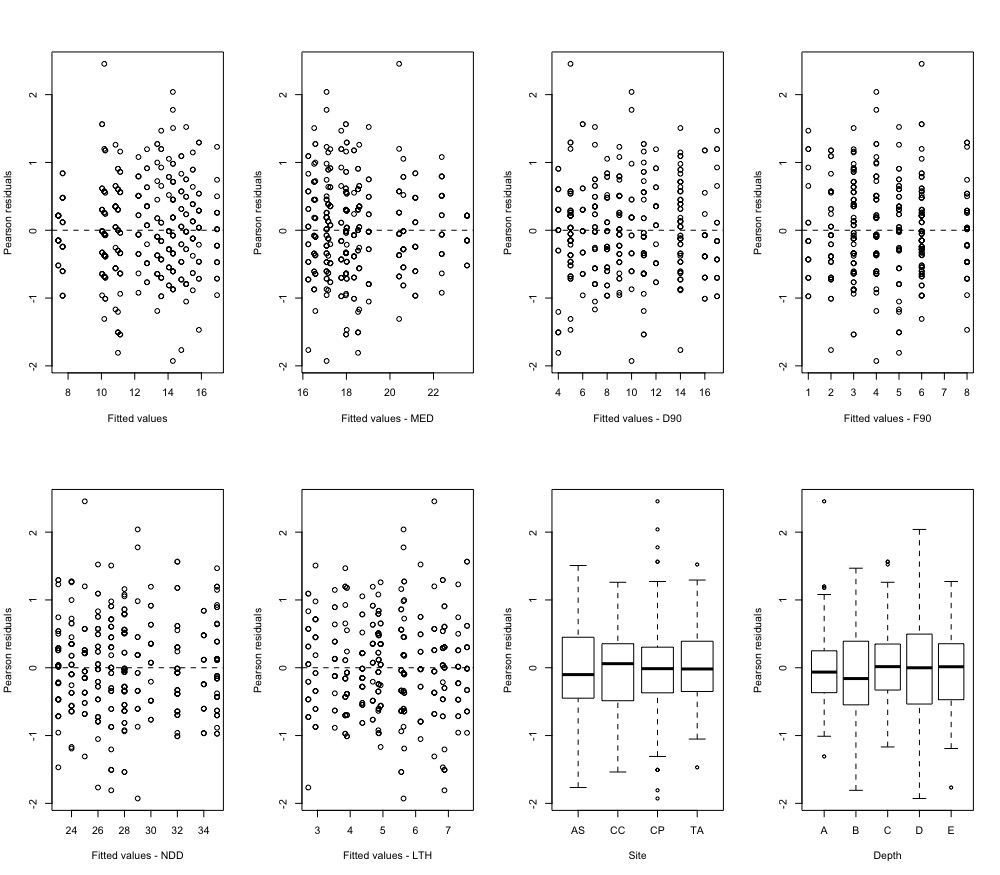
**

**Evenness model selection. “x” = interaction; “s” = smoother. Distribution: Gaussian. In bold the model selected. The model order follows an increasing AIC.**

| Model | Df | AIC | ∆AIC | Dev |
| --- | --- | --- | --- | --- |
| **evenness ~ factor(Site) + s(MED, by = Site)** | **17.81** | **-2316.95** | **0** | **25.6** |
| evenness ~ factor(Site) + s(MED, by = Site) + s(LTH) | 15.02 | -2316.38 | 1 | 25.4 |
| evenness ~ factor(Site) + s(MED, by = Site) + s(F90) + s(LTH) | 15.98 | -2314.82 | 2.23 | 25.50 |
| evenness ~ factor(Site) + s(MED, by = Site) + s(F90) + s(LTH, by= Site) | 16.85 | -2314.15 | 2.90 | 25.70 |
| evenness ~ factor(Site) + s(MED) + s(F90) + s(LTH) | 16.24 | -2314.13 | 2.92 | 25.30 |
| evenness ~ factor(Site) + s(MED, by = Depth) + s(F90) + s(LTH) | 17.58 | -2314.11 | 2.94 | 26.00 |
| evenness ~ factor(Site) + s(MED, by = Site) + s(F90) + s(LTH, by= Depth) | 17.97 | -2313.81 | 3.24 | 26.10 |
| evenness ~ factor(Site) + s(MED, by = Site) + s(F90) | 17.29 | -2313.76 | 3.29 | 25.80 |
| evenness ~ factor(Site) + s(MED, by = Site) + s(F90, by=Site) + s(LTH) | 17.57 | -2313.20 | 3.85 | 26.00 |
| evenness ~ factor(Site) + s(MED, by = Site) + s(F90, by=Depth) + s(LTH) | 17.85 | -2312.53 | 4.52 | 25.80 |
| evenness ~ factor(Site) + s(MED) + s(F90) + s(D90) + s(NDD) + s(LTH) | 17.53 | -2312.10 | 4.95 | 25.60 |
| evenness ~ factor(Site) + s(MED) + s(F90) + s(NDD) + s(LTH) | 17.77 | -2311.59 | 5.46 | 25.50 |
| evenness ~ factor(Site) + s(MED) + s(F90) + s(D90) | 17.74 | -2311.23 | 5.82 | 25.50 |
| evenness ~ factor(Site) + s(MED, by = Depth) | 18.83 | -2310.93 | 6.12 | 25.80 |
| evenness ~ factor(Site) + s(MED) + s(F90) + s(D90) + s(NDD) | 18.79 | -2310.76 | 6.29 | 25.80 |
| evenness ~ factor(Site) + s(MED) + s(F90) | 19.65 | -2310.17 | 6.88 | 26.00 |
| evenness ~ factor(Site) + s(MED) + s(LTH) | 17.30 | -2307.07 | 9.98 | 24.60 |
| evenness ~ factor(Site) + s(MED) | 11.78 | -2290.26 | 26.79 | 19.10 |
| evenness ~ factor(Site) + s(MED) + s(F90) | 13.24 | -2289.52 | 27.53 | 19.60 |
| evenness ~ factor(Site) | 5.00 | -2272.15 | 44.91 | 12.40 |
| evenness ~ factor(Site) + factor(Depth) | 9.00 | -2268.54 | 48.51 | 13.40 |

**Evenness model validation
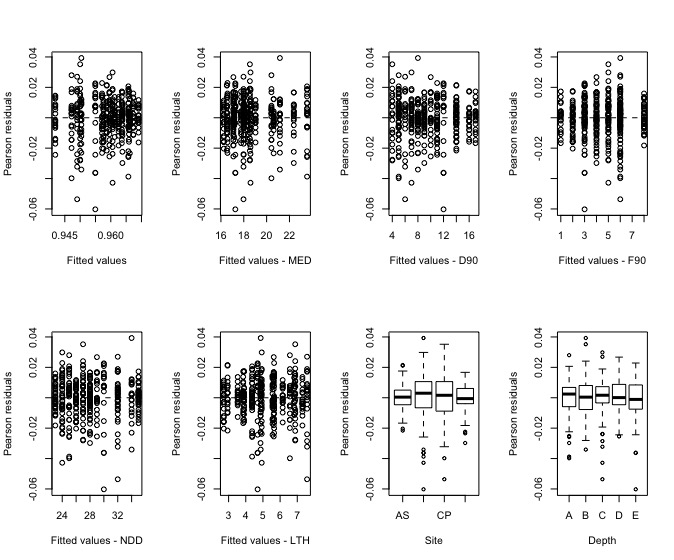
**

**Corallinales model selection. “x” = interaction; “s” = smoother. Distribution: Negative binomial. In bold the model selected. The model order follows an increasing AIC.**

| **Model** | **AIC** | **∆AIC** | **df** | **Dev** |
| --- | --- | --- | --- | --- |
| **CorallinalesTot ~ factor(Site) + factor(Depth) + s(MED) + s(D90xDepth)** | **2700.23** | **0.00** | **16.56** | **26.7** |
| CorallinalesTot ~ factor(Site) + factor(Depth)+ s(MED) + s(D90xDepth) + s(NDD) | 2700.59 | 0.35 | 18.40 | 27.2 |
| CorallinalesTot ~ factor(Site) + factor(Depth) + s(MEDxDepth) + s(D90xDepth) + s(F90) + s(NDD) | 2701.83 | 1.59 | 19.23 | 27.3 |
| CorallinalesTot ~ factor(Site) + factor(Depth) + s(MED + s(D90) + s(F90) + s(NDD) | 2702.03 | 1.79 | 19.13 | 27.2 |
| CorallinalesTot ~ factor(Site) + factor(Depth) + s(MED) + s(D90) + s(F90) + s(NDD) + s(LTH) | 2702.60 | 2.37 | 19.41 | 27.2 |
| CorallinalesTot ~ factor(Site) + factor(Depth) + s(MEDxSite) + s(D90) + s(F90) + s(NDD) | 2702.71 | 2.47 | 19.42 | 27.2 |
| CorallinalesTot ~ factor(Site) + factor(Depth) + s(MEDxDepth) + s(D90) + s(F90xSite) + s(NDD) | 2702.98 | 2.74 | 20.00 | 27.4 |
| CorallinalesTot ~ factor(Site) + factor(Depth) + s(MEDxDepth) + s(D90) + s(F90) + s(NDDxSite) | 2703.39 | 3.16 | 20.01 | 27.3 |
| CorallinalesTot ~ factor(Site) + factor(Depth) + s(MEDxDepth) + s(D90xSite) + s(F90) + s(NDD) | 2703.49 | 3.26 | 19.76 | 27.2 |
| CorallinalesTot ~ factor(Site) + factor(Depth) + s(MED) + s(D90) + s(F90) | 2704.17 | 3.94 | 19.00 | 26.8 |
| CorallinalesTot ~ factor(Site) + factor(Depth) + s(MED:Depth) + s(D90) + s(F90) + s(NDD) | 2704.24 | 4.01 | 20.45 | 27.3 |
| CorallinalesTot ~ factor(Site) + factor(Depth) + s(MED) + s(D90xDepth) + s(F90) + s(NDDxDepth) | 2704.60 | 4.36 | 21.00 | 27.5 |
| CorallinalesTot ~ factor(Site) + factor(Depth) + s(MEDxDepth) + s(D90) + s(F90xDepth) + s(NDD) | 2704.60 | 4.36 | 21.00 | 27.5 |
| CorallinalesTot ~ factor(Site) + factor(Depth) + s(MED) + s(D90) | 2704.81 | 4.58 | 18.75 | 26.6 |
| CorallinalesTot ~ factor(Site) + factor(Depth) + s(MED) | 2715.26 | 15.03 | 17.07 | 24.2 |
| CorallinalesTot ~ factor(Site) + factor(Depth) | 2759.57 | 59.34 | 9.00 | 12.4 |
| CorallinalesTot ~ factor(Site) | 2782.05 | 81.82 | 5.00 | 5.91 |

**Corallinales model validation
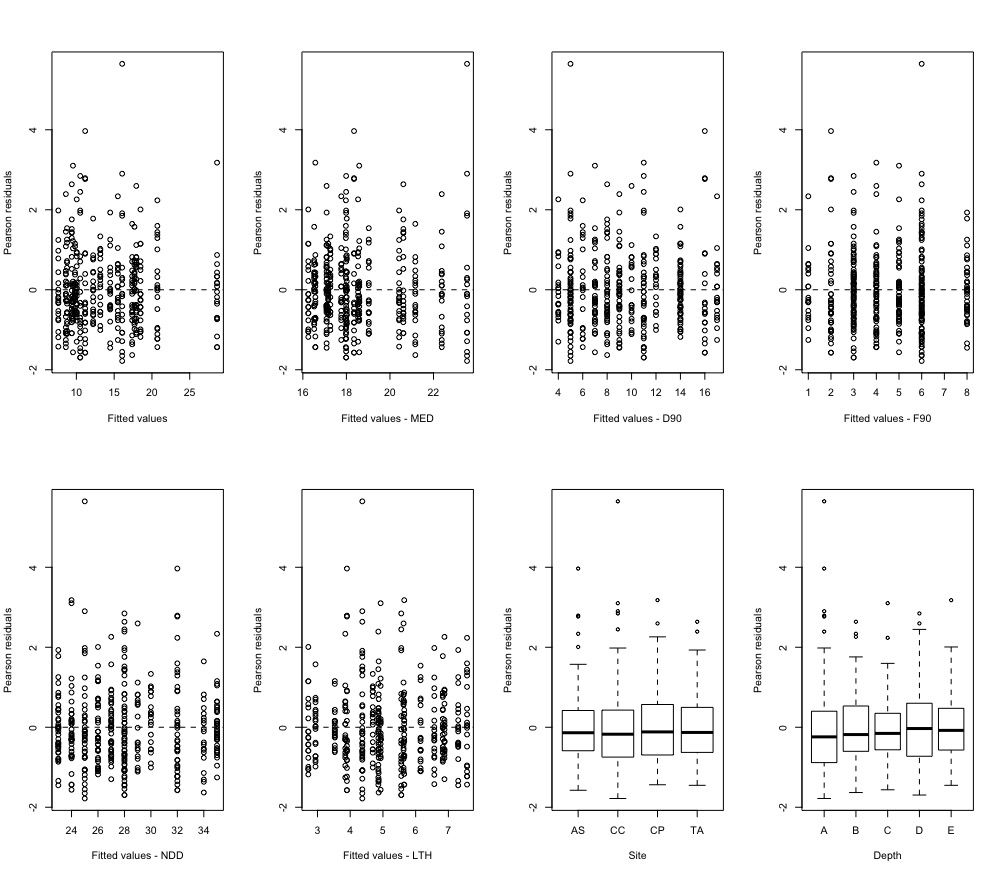
**

**Green algae model selection. “x” = interaction; “s” = smoother. Distribution: Negative binomial. In bold the model selected. The model order follows an increasing AIC.**

| Model | Df | AIC | ∆AIC | Dev |
| --- | --- | --- | --- | --- |
| GreenAlgae ~ factor(Site) + s(MEDxSite) | 15.88 | 1834.27 | 0.00 | 65.4 |
| GreenAlgae ~ factor(Site) + factor(Depth) + s(MEDxSite) | 17.08 | 1834.33 | 0.06 | 65.6 |
| GreenAlgae ~factor(Site) + factor(Depth) + s(MEDxSite) + s(LTH) | 18.13 | 1835.08 | 0.81 | 65.8 |
| GreenAlgae ~factor(Site) + factor(Depth) + s(MEDxSite) + s(NDD) | 17.70 | 1835.72 | 1.46 | 65.6 |
| GreenAlgae ~ factor(Site) + factor(Depth) + s(MEDxSite) + s(F90) | 17.95 | 1835.90 | 1.63 | 65.6 |
| GreenAlgae ~ factor(Site) + factor(Depth) + s(MEDxSite) + s(D90) | 18.05 | 1836.08 | 1.81 | 65.6 |
| GreenAlgae ~ factor(Depth) + s(MEDxSite) | 18.55 | 1837.12 | 2.86 | 65.6 |
| GreenAlgae ~ factor(Site) + factor(Depth) + s(MEDxDepth) | 19.89 | 1837.81 | 3.54 | 65.8 |
| GreenAlgae ~ factor(Site) + factor(Depth) + s(MED) | 17.96 | 1838.39 | 4.13 | 65.4 |
| GreenAlgae ~ s(MEDxSite) | 19.13 | 1838.70 | 4.43 | 65.6 |
| GreenAlgae ~ factor(Site) + factor(Depth) + s(MED) + s(D90) + s(F90) + s(NDD) + s(LTH) | 20.93 | 1839.93 | 5.66 | 65.8 |
| GreenAlgae ~ factor(Site) + factor(Depth) + s(MED) + s(D90) + s(F90) | 20.14 | 1840.07 | 5.80 | 65.6 |
| GreenAlgae ~ factor(Site) + factor(Depth) + s(MED) + s(D90) + s(F90) + s(NDD) | 20.59 | 1840.29 | 6.02 | 65.7 |
| GreenAlgae ~ factor(Site) + factor(Depth) + s(MED) + s(D90) | 20.01 | 1842.58 | 8.32 | 65.4 |
| GreenAlgae ~ factor(Site) + factor(Depth) | 9.00 | 1978.12 | 143.85 | 47.5 |
| GreenAlgae ~ factor(Site) | 5.00 | 2864.46 | 1030.20 | 25.0 |

**Green Algae model validation
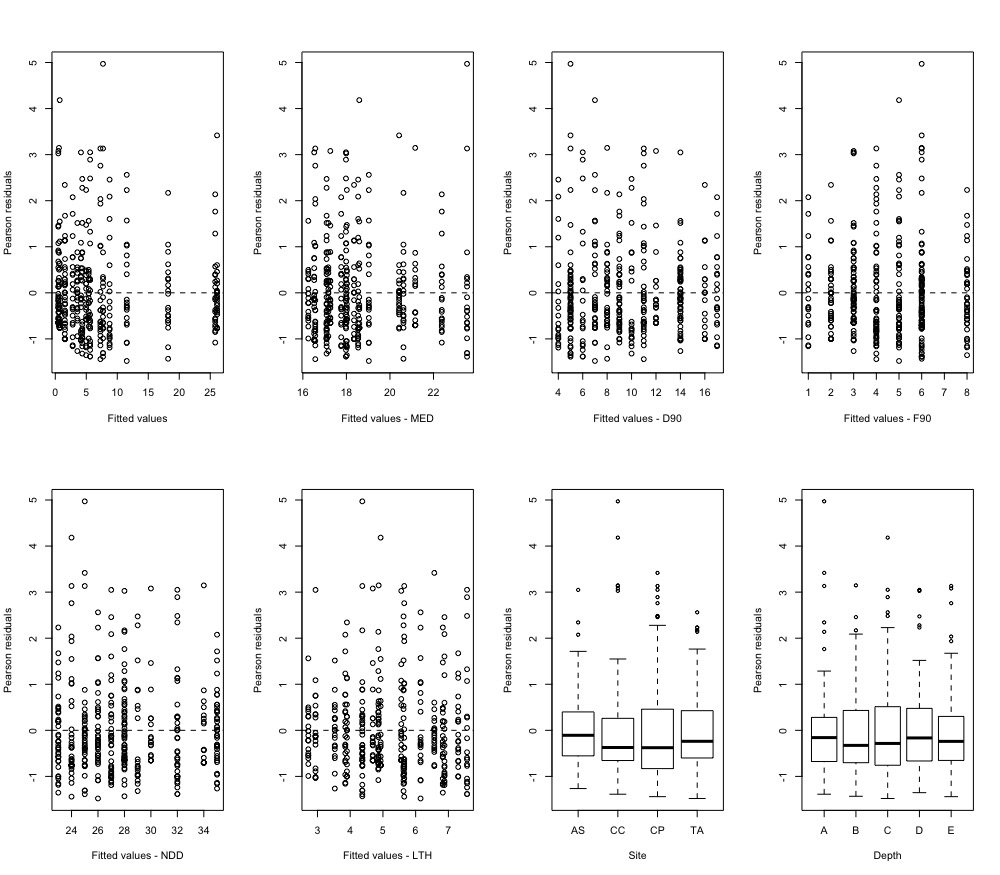
**

**Red algae model selection. “x” = interaction; “s” = smoother. Distribution: Negative binomial. In bold the model selected. The model order follows an increasing AIC.**

| Model | Df | AIC | ∆AIC | Dev |
| --- | --- | --- | --- | --- |
| **RedAlgae ~ factor(Site)+ factor(Depth) + s(D90xSite) + s(F90)** | **16.52** | **3315.72** | **0.00** | **29.7** |
| RedAlgae ~ factor(Site) + factor(Depth) + s(MEDxSite) + s(D90xSite) + s(F90) | 18.00 | 3316.81 | 1.09 | 30.0 |
| RedAlgae ~ factor(Site) + s(MEDxSite) + s(D90xSite) + s(F90) | 18.28 | 3318.03 | 2.31 | 29.9 |
| RedAlgae ~ factor(Site) + factor(Depth) + s(MEDxSite)+ s(F90) | 18.48 | 3318.14 | 2.42 | 30.0 |
| RedAlgae ~ factor(Site) + factor(Depth) + s(MEDxSite) + s(D90xSite) + s(F90) + s(NDD) | 19.00 | 3318.15 | 2.43 | 30.1 |
| RedAlgae ~ factor(Site) + factor(Depth) + s(MEDxSite) + s(D90) + s(F90) + s(NDD) | 19.25 | 3318.96 | 3.24 | 30.1 |
| RedAlgae ~ factor(Depth) + s(MEDxSite) + s(D90xSite) + s(F90) | 18.88 | 3319.11 | 3.39 | 29.9 |
| RedAlgae ~ factor(Site) + factor(Depth) + s(MEDxDepth) + s(D90) + s(F90) + s(NDD) | 19.51 | 3319.33 | 3.61 | 30.1 |
| RedAlgae ~ factor(Site)+ factor(Depth) + s(D90xSite) | 16.78 | 3319.66 | 3.94 | 29.2 |
| RedAlgae ~ factor(Site) + factor(Depth) + s(MEDxSite) + s(D90xSite) | 18.82 | 3319.93 | 4.21 | 29.8 |
| RedAlgae ~ factor(Site) + factor(Depth) + s(MED) + s(D90) + s(F90) + s(NDD) | 18.92 | 3320.13 | 4.41 | 29.8 |
| RedAlgae ~ factor(Site) + factor(Depth) + s(MED) | 17.25 | 3321.01 | 5.29 | 29.2 |
| RedAlgae ~ factor(Site) + factor(Depth) + s(MED) + s(D90) | 18.49 | 3321.24 | 5.52 | 29.5 |
| RedAlgae ~ factor(Site) + factor(Depth) + s(MEDxSite) + s(D90xSite) + s(NDD) | 19.85 | 3321.36 | 5.64 | 29.9 |
| RedAlgae ~ factor(Site) + factor(Depth) + s(MED) + s(D90) + s(F90) + s(NDD) + s(LTH) | 19.67 | 3321.52 | 5.80 | 29.8 |
| RedAlgae ~ factor(Site) + factor(Depth) + s(MEDxSite) + s(D90xDepth) + s(F90) + s(NDD) | 21.00 | 3321.79 | 6.07 | 30.1 |
| RedAlgae ~ factor(Site) + factor(Depth) + s(MED) + s(D90) + s(F90) | 18.66 | 3322.52 | 6.80 | 29.4 |
| RedAlgae ~ factor(Site) + factor(Depth) + s(MEDxSite) + s(D90xSite) + s(F90xSite) + s(NDD) | 22.00 | 3323.75 | 8.03 | 30.1 |
| RedAlgae ~ factor(Site) + factor(Depth) +s(MEDxSite) + s(D90xSite) + s(F90xDepth) + s(NDD) | 22.00 | 3323.75 | 8.03 | 30.1 |
| RedAlgae ~ factor(Site) + factor(Depth) + s(MEDxSite) + s(D90xSite) + s(F90) + s(NDDx Site) | 22.00 | 3323.75 | 8.03 | 30.1 |
| RedAlgae ~ factor(Site) + factor(Depth) + s(MEDxSite) + s(D90xSite) + s(F90) + s(NDDxDepth) | 22.00 | 3323.75 | 8.03 | 30.1 |
| RedAlgae ~ factor(Site)+ factor(Depth) + s(F90) | 14.91 | 3338.47 | 22.75 | 25.8 |
| RedAlgae ~ factor(Site) + factor(Depth) | 9.00 | 3370.89 | 55.17 | 18.5 |
| RedAlgae ~ factor(Site) | 5.00 | 3374.38 | 58.67 | 16.5 |

**Red Algae model validation
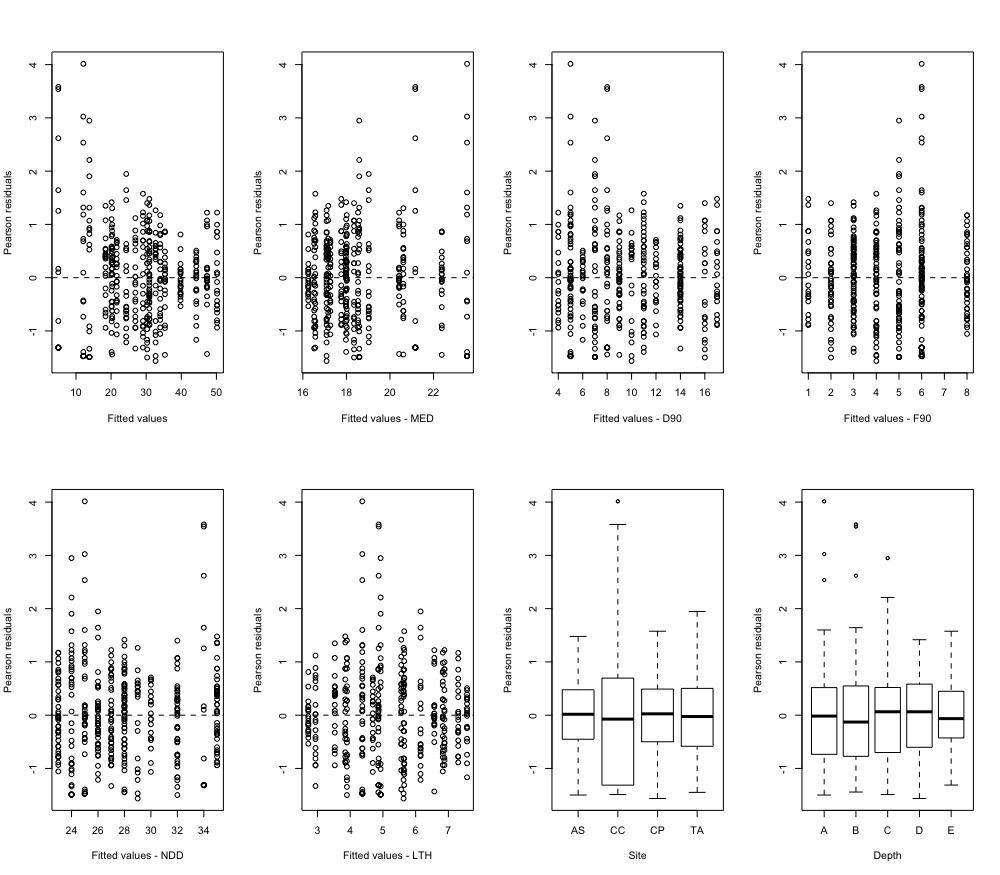
**

**Turf model selection. “x” = interaction; “s” = smoother. Distribution: Negative binomial. In bold the model selected. The model order follows an increasing AIC.**

| Model | Df | AIC | ∆AIC | Dev |
| --- | --- | --- | --- | --- |
| **Turf ~ factor(Site) + factor(Depth)+ s(D90xDepth) + s(NDD)** | **16.98** | **2976.65** | **0.00** | **34.8** |
| Turf ~ factor(Site) + factor(Depth)+ s(D90xDepth) + s(F90) + s(NDD) | 17.24 | 2976.75 | 0.10 | 34.8 |
| Turf ~ factor(Site) + factor(Depth) + s(MED) + s(D90xDepth) + s(F90) + s(NDD) | 18.24 | 2978.17 | 1.53 | 34.9 |
| Turf ~ factor(Site) + factor(Depth) + s(MED) + s(D90) + s(F90) + s(NDD) | 19.08 | 2979.86 | 3.21 | 34.9 |
| Turf ~ factor(Site) + factor(Depth) + s(MEDxDepth) + s(D90) + s(F90) + s(NDD) | 19.57 | 2980.68 | 4.03 | 34.9 |
| Turf ~ factor(Site) + factor(Depth) + s(MED) + s(D90) + s(F90) + s(NDD) + s(LTH) | 19.04 | 2980.74 | 4.09 | 34.8 |
| Turf ~ factor(Site) + factor(Depth) + s(MED) + s(D90xSite) + s(F90) + s(NDD) | 19.69 | 2981.32 | 4.67 | 34.9 |
| Turf ~ factor(Site) + factor(Depth) + s(MEDxSite) + s(D90) + s(F90) + s(NDD) | 20.19 | 2981.54 | 4.89 | 35.0 |
| Turf ~ factor(Site) + factor(Depth) + s(MED) + s(D90xDepth) + s(F90xDepth) + s(NDD) | 21.00 | 2982.29 | 5.64 | 35.1 |
| Turf ~ factor(Site) + factor(Depth) + s(MED) + s(D90xDepth) + s(F90) + s(NDDxDepth) | 21.00 | 2982.29 | 5.64 | 35.1 |
| Turf ~ factor(Site) + factor(Depth) + s(MED) + s(D90xDepth) + s(F90) + s(NDDxSite) | 20.97 | 2982.40 | 5.75 | 35.1 |
| Turf ~ factor(Site) + factor(Depth) + s(MED) + s(D90xDepth) + s(F90xSite) + s(NDD) | 20.92 | 2982.41 | 5.76 | 35.1 |
| Turf ~ factor(Site) + factor(Depth)+ s(F90) + s(NDD) | 16.44 | 2982.53 | 5.78 | 33.7 |
| Turf ~ factor(Site) + factor(Depth) + s(MED) + s(D90) + s(NDD) | 17.83 | 2984.15 | 7.50 | 33.9 |
| Turf ~ factor(Site) + factor(Depth) + s(D90) + s(NDD) | 16.46 | 2984.44 | 7.80 | 33.5 |
| Turf ~ factor(Site) + factor(Depth)+ s(NDD) | 15.12 | 2985.97 | 9.32 | 32.8 |
| Turf ~ factor(Site) + factor(Depth)+ s(D90xDepth) + s(F90) | 17.58 | 2986.79 | 10.15 | 33.4 |
| Turf ~ factor(Site) + factor(Depth) + s(MED) + s(D90) | 17.61 | 2987.38 | 10.73 | 33.4 |
| Turf ~ factor(Site) + factor(Depth) + s(MED) + s(D90) + s(F90) | 17.53 | 2987.58 | 10.93 | 33.3 |
| Turf ~ factor(Site) + factor(Depth)+ s(D90xDepth) | 15.81 | 2990.84 | 14.20 | 32.3 |
| Turf ~ factor(Site) + factor(Depth) + s(MED) | 12.10 | 3003.23 | 26.58 | 29.2 |
| Turf ~ factor(Site) + factor(Depth) | 9.00 | 3008.36 | 31.71 | 27.3 |
| Turf~ factor(Site) | 5.00 | 3029.06 | 52.42 | 22.5 |

**Turf model validation
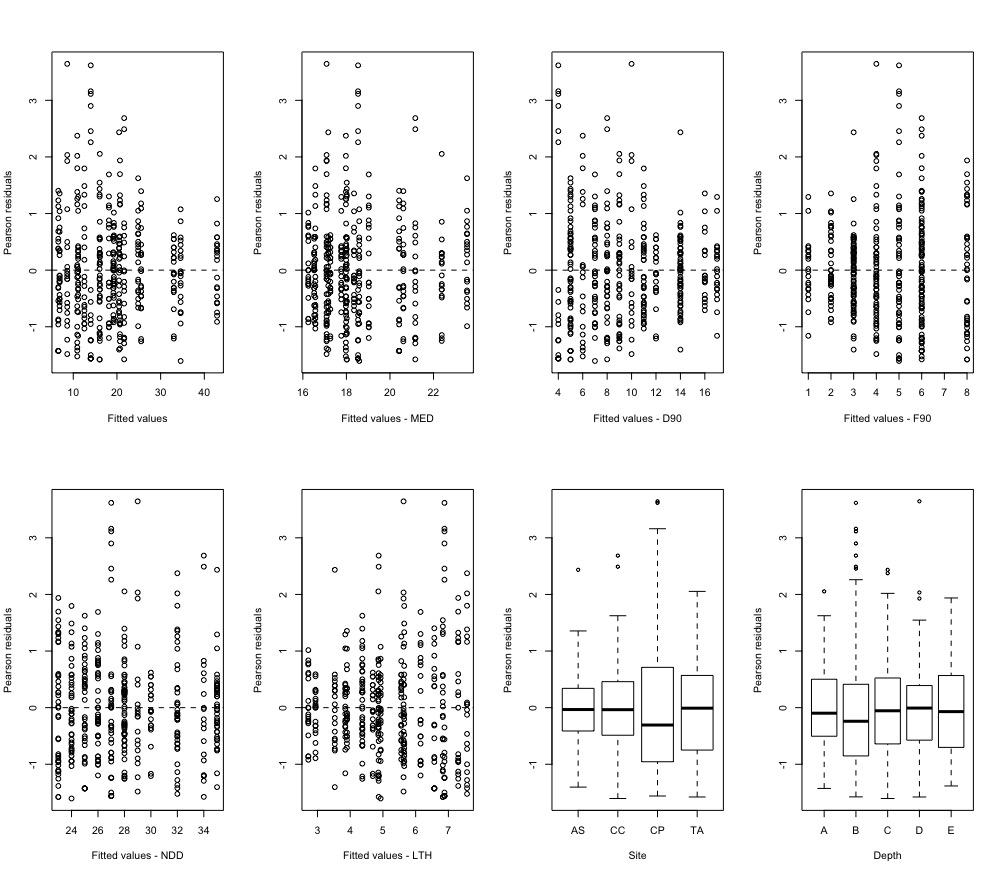
**

**Briozoans model selection. “x” = interaction; “s” = smoother. Distribution: Negative binomial. In bold the model selected. The model order follows an increasing AIC.**

| Model | Df | AIC | ∆AIC | Dev |
| --- | --- | --- | --- | --- |
| **Briozoans ~ factor(Site) + factor(Depth) + s(MEDxDepth) + s(D90xSite) + s(NDD)** | **19.25** | **1768.82** | **0.00** | **38.7** |
| Briozoans ~ factor(Site) + factor(Depth) + s(MEDxDepth) + s(D90xSite) + s(F90) + s(NDD) | 20.09 | 1770.47 | 1.65 | 38.7 |
| Briozoans ~ factor(Site) + factor(Depth) + s(MEDxDepth) + s(D90) + s(F90) + s(NDD) | 20.39 | 1770.98 | 2.15 | 38.7 |
| Briozoans ~ factor(Site) + factor(Depth) + s(MED) + s(D90) + s(F90) + s(NDD) | 19.58 | 1771.03 | 2.21 | 38.5 |
| Briozoans ~ factor(Site) + factor(Depth) + s(MEDx Depth) + s(D90xSite) | 20.59 | 1771.34 | 2.52 | 38.7 |
| Briozoans ~ factor(Site) + factor(Depth) + s(MEDxDepth) + s(D90xDepth) + s(F90) + s(NDD) | 21.00 | 1771.43 | 2.61 | 38.8 |
| Briozoans ~ factor(Site) + factor(Depth) + s(D90xSite) + s(NDD) | 20.86 | 1771.69 | 2.87 | 38.8 |
| Briozoans ~ factor(Site) + factor(Depth) + s(MEDxDepth) + s(NDD) | 20.41 | 1771.88 | 3.06 | 38.6 |
| Briozoans ~ factor(Site) + factor(Depth) + s(MED) + s(D90) + s(F90) + s(LTH) | 18.50 | 1772.06 | 3.24 | 38.0 |
| Briozoans ~ factor(Site) + factor(Depth) + s(MEDxSite) + s(D90) + s(F90) + s(NDD) | 20.82 | 1772.15 | 3.33 | 38.7 |
| Briozoans ~ factor(Site) + factor(Depth) + s(MEDxDepth) + s(D90xSite) + s(F90xSite) + s(NDD) | 22.00 | 1773.41 | 4.58 | 38.8 |
| Briozoans ~ factor(Site) + factor(Depth) + s(MEDxDepth) + s(D90xSite) + s(F90xDepth) + s(NDD) | 22.00 | 1773.41 | 4.58 | 38.8 |
| Briozoans ~ factor(Site) + factor(Depth) + s(MEDxDepth) + s(D90xSite) + s(F90) + s(NDDxSite) | 22.00 | 1773.41 | 4.58 | 38.8 |
| Briozoans ~ factor(Site) + factor(Depth) + s(MEDxDepth) + s(D90xSite) + s(F90) + s(NDDxDepth) | 22.00 | 1773.41 | 4.58 | 38.8 |
| Briozoans ~ factor(Site) + factor(Depth) + s(MED) + s(D90) + s(F90) | 16.23 | 1781.64 | 12.82 | 36.0 |
| Briozoans ~ factor(Site) + factor(Depth) + s(MED) + s(D90) | 15.59 | 1786.20 | 17.37 | 35.1 |
| Briozoans~ factor(Site) + factor(Depth) + s(MED) | 14.11 | 1789.52 | 20.70 | 34.2 |
| Briozoans ~ factor(Site) + factor(Depth) | 9.00 | 1891.12 | 122.30 | 15.5 |
| Briozoans ~ factor(Site) | 5.00 | 1956.10 | 187.28 | 1.31 |

**Briozoans model validation
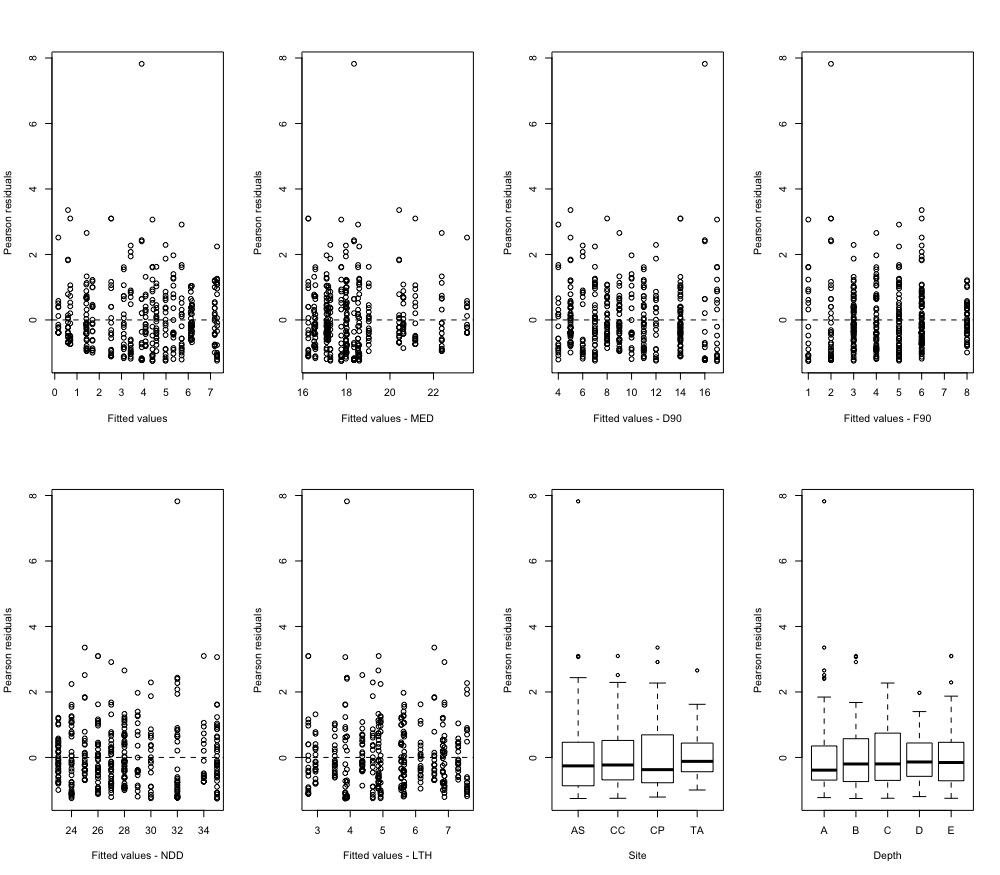
**

**Sponges model selection. “x” = interaction; “s” = smoother. Distribution: Negative binomial. In bold the model selected. The model order follows an increasing AIC.**

| Model | Df | AIC | ∆AIC | Dev |
| --- | --- | --- | --- | --- |
| **SpongesTot ~ factor(Site) + factor(Depth) + s(D90xSite) + s(F90)** | **16.60** | **2482.03** | **0.00** | **29.0** |
| SpongesTot ~ factor(Site) + factor(Depth) + s(MEDxDepth) + s(D90xSite) + s(F90) | 20.00 | 2482.59 | 0.56 | 29.9 |
| SpongesTot ~ factor(Site) + factor(Depth) + s(MEDxDepth) + s(F90) | 19.93 | 2482.84 | 0.81 | 29.9 |
| SpongesTot ~ factor(Site) + s(D90xSite) + s(F90) | 16.80 | 2482.87 | 0.84 | 28.9 |
| SpongesTot ~ factor(Site) + factor(Depth) + s(MEDxDepth) + s(D90) + s(F90) | 20.06 | 2482.94 | 0.91 | 29.9 |
| SpongesTot ~ factor(Site) + factor(Depth) + s(MEDxDepth) + s(D90xSite) | 19.74 | 2482.97 | 0.94 | 29.8 |
| SpongesTot ~ factor(Site) + factor(Depth) + s(MEDxDepth) + s(D90xDepth) + s(F90) | 20.01 | 2483.08 | 1.05 | 29.9 |
| SpongesTot ~ factor(Depth) + s(D90xSite) + s(F90) | 16.65 | 2483.71 | 1.68 | 28.7 |
| SpongesTot ~ factor(Site) + factor(Depth) + s(MED) + s(D90) + s(F90) | 18.47 | 2484.17 | 2.14 | 29.2 |
| SpongesTot ~ factor(Site) + factor(Depth) + s(MED) + s(D90) + s(F90) + s(NDD) + s(LTH) | 20.42 | 2484.92 | 2.89 | 29.7 |
| SpongesTot ~ factor(Site) + factor(Depth) + s(MED) + s(D90) + s(NDD) | 19.89 | 2485.11 | 3.08 | 29.5 |
| SpongesTot ~ factor(Site) + factor(Depth) + s(MEDxSite) + s(D90) + s(F90) | 18.59 | 2485.16 | 3.13 | 29.1 |
| SpongesTot ~ factor(Site) + factor(Depth) + s(MED) + s(D90) + s(F90) + s(NDD) | 20.37 | 2485.19 | 3.16 | 29.6 |
| SpongesTot ~ factor(Site) + factor(Depth) + s(MED) + s(D90) | 20.39 | 2485.68 | 3.65 | 29.6 |
| SpongesTot ~ factor(Site) + factor(Depth) + s(D90xSite) | 17.75 | 2485.72 | 3.69 | 28.7 |
| SpongesTot ~ factor(Site) + factor(Depth) + s(MED) + s(D90) + s(LTH) | 20.13 | 2486.07 | 4.04 | 29.4 |
| SpongesTot ~ factor(Site) + factor(Depth) + s(MEDxDepth) + s(D90xSite) + s(F90xSite) | 22.00 | 2486.21 | 4.18 | 30.0 |
| SpongesTot ~ factor(Site) + factor(Depth) + s(MEDxDepth) + s(D90xSite) + s(F90xDepth) | 22.00 | 2486.21 | 4.18 | 30.0 |
| SpongesTot ~ factor(Site) + factor(Depth) + s(MED) | 17.35 | 2509.25 | 27.22 | 24.7 |
| SpongesTot ~ factor(Site) + factor(Depth) + s(F90) | 14.97 | 2511.20 | 29.18 | 23.6 |
| SpongesTot ~ factor(Site) + factor(Depth) | 9.00 | 2563.24 | 81.21 | 12.1 |
| SpongesTot ~ factor(Site) | 5.00 | 2578.78 | 96.75 | 7.52 |

**Sponges tot model validation
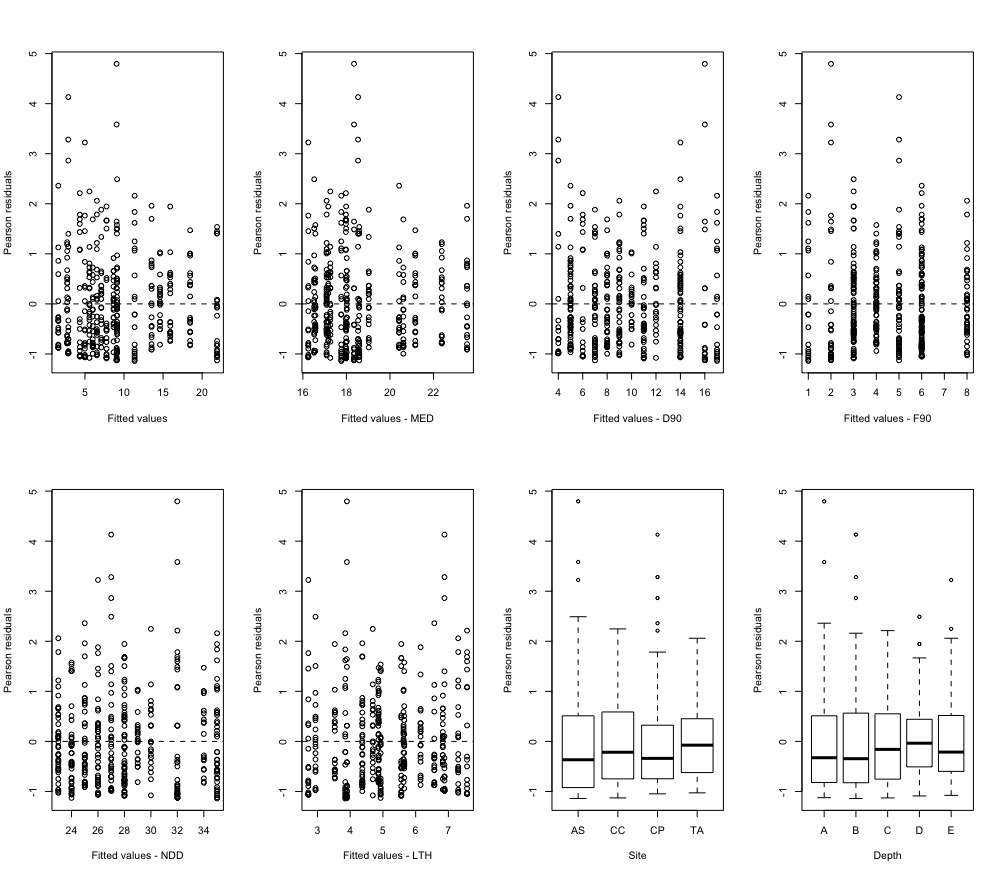
**
